# Whole genome sequencing reveals signals of adaptive admixture in Creole cattle

**DOI:** 10.1038/s41598-023-38774-7

**Published:** 2023-07-27

**Authors:** Slim Ben-Jemaa, Gabriele Adam, Mekki Boussaha, Philippe Bardou, Christophe Klopp, Nathalie Mandonnet, Michel Naves

**Affiliations:** 1 INRAE, ASSET, 97170 Petit-Bourg, France; 2 Laboratoire des Productions Animales et Fourragères, Institut National de la Recherche Agronomique de Tunisie, Université de Carthage, 2049 Ariana, Tunisia; 3 AgroParisTech, GABI, INRAE, Université Paris-Saclay, 78350 Jouy-en-Josas, France; 4 GenPhySE, Ecole Nationale Vétérinaire de Toulouse (ENVT), INRA, Université de Toulouse, 24 Chemin de Borde Rouge, 31320 Castanet-Tolosan, France; 5 Sigenae, INRAE, 24 Chemin de Borde Rouge, 31320 Castanet-Tolosan, France; 6 Genotoul Bioinfo, BioInfoMics, MIAT UR875, Sigenae, INRAE, Castanet-Tolosan, France

**Keywords:** Computational biology and bioinformatics, Genetics, Molecular biology

## Abstract

The Creole cattle from Guadeloupe (GUA) are well adapted to the tropical environment. Its admixed genome likely played an important role in such adaptation. Here, we sought to detect genomic signatures of selection in the GUA genome. For this purpose, we sequenced 23 GUA individuals and combined our data with sequenced genomes of 99 animals representative of European, African and indicine groups. We detect 17,228,983 single nucleotide polymorphisms (SNPs) in the GUA genome, providing the most detailed exploration, to date, of patterns of genetic variation in this breed. We confirm the higher level of African and indicine ancestries, compared to the European ancestry and we highlight the African origin of indicine ancestry in the GUA genome. We identify five strong candidate regions showing an excess of indicine ancestry and consistently supported across the different detection methods. These regions encompass genes with adaptive roles in relation to immunity, thermotolerance and physical activity. We confirmed a previously identified horn-related gene, *RXFP2*, as a gene under strong selective pressure in the GUA population likely owing to human-driven (socio-cultural) pressure. Findings from this study provide insight into the genetic mechanisms associated with resilience traits in livestock.

## Introduction

One of the major consequences of long-distance human migrations is the displacement of domestic animals into new environments, thus putting a strong selective pressure on the genome of these animals over a brief period. One example is the introduction of cattle to the western hemisphere. New World Creole cattle were first brought from the Iberian Peninsula by Spanish colonists since the second expedition of Christopher Columbus in the late fifteenth century. Creole cattle breeds have then undergone a rapid expansion throughout the American continent^1^. Subsequently, between the sixteenth and eighteenth centuries, West African cattle are thought to have entered the Caribbean and Brazil, presumably as a consequence of slave trade routes^2,3^. Genetic evidence also points to a West African influence on Creole cattle^4,5^. Around the middle of the nineteenth century, several other European cattle breeds were brought in large numbers to the Americas. Later on, during the beginning of the twentieth century, *Bos indicus* were imported from India to improve the adaptability of local populations in tropical areas of the Americas through extensive crossbreeding^3,6^.

The Creole cattle in the Guadeloupe island (GUA) is an admixed breed. Published estimates of ancestry proportions of this population indicate 26%, 36% and 38% of European taurine (EUT), African taurine (AFT) and indicine (IND) ancestries in the GUA genome, respectively^7^. The GUA population is well adapted to tropical environment and to the conditions of the local production systems characterized by nutrient-poor pastures. This is exemplified by low feed requirement, heat tolerance and by the ability to make use of poor-quality forage efficiently to have an acceptable body weight gain^8^. The GUA population also appears particularly resistant to local parasitic and infectious diseases^9^.

Elucidating the genetic architecture of adaptation is becoming an increasingly relevant topic in animal genetics. Indeed, it is expected that innovative breeding schemes will include heritable resilience biomarkers to overcome the foreseeable adverse impacts of climate change characterized by increasing temperatures, the expansion into new areas of invasive parasites and possibly the degradation of forage production and quality. An increasingly number of studies have addressed the effect of admixture and introgression in cattle adaptation to new environmental challenges^10–12^. These studies benefited from ongoing advancements in genomic technology and the development of improved statistical and computational methods to identify signatures of selection in cattle genome, that is genomic regions that appear to be shaped by selection.

Owing to its three-way admixture and its long-term isolation under extreme environmental conditions, the genome of the Creole cattle from Guadeloupe offers a unique opportunity to study admixture-enabled adaptation.

There are only a few studies that aimed at detecting selection signatures in Creole cattle^6,7,13^. The results from these studies point to candidate regions linked to various molecular processes underlying tropical adaptation including slick hair coat, DNA repair processes^6^, immune response and adaptation to warm conditions in male and female reproductive functions^7,13^.

A previous study provided a first insight into footprints of selection in GUA cattle using the Illumina BovineSNP50 chip assay^7^. To expand our understanding of the genomic architecture of local adaptation in this population, we leverage whole-genome sequence data from 23 GUA animals and genomes of 99 cattle individuals from various origins. Our objective was to use high-resolution genomic data and consistency of signals among different methods based on the excess of haplotype homozygosity, differences in allele frequencies and excess ⁄ deficiency of local ancestry to identify new, strong candidate regions under selection in the GUA genome.

## Results

### Sequencing and detection of variation

A total of 7,543,644,154 reads were generated after sequencing the complete genome of the 23 Creole cattle samples. The reads were aligned to the latest *Bos Taurus* reference genome (ARS-UCD1.2) with an average alignment rate of 95.7% (ranging between 94.93 and 96.51%) and an average depth of 16.35 (min depth = 9.3; max depth = 23.77) (Supplementary Table S1). We identified a total of 17,228,983 filtered SNPs in the 23 GUA individuals (Supplementary Table S2). Functional annotation revealed that the large majority of SNPs are located within intronic (48%) and intergenic regions (41.5%) while exons accounted for 0.89% of total SNPs including 928 nonsense and 87,621 missense mutations (Supplementary Table S3).

### Genetic diversity and ROH detection

**Figure 1:**
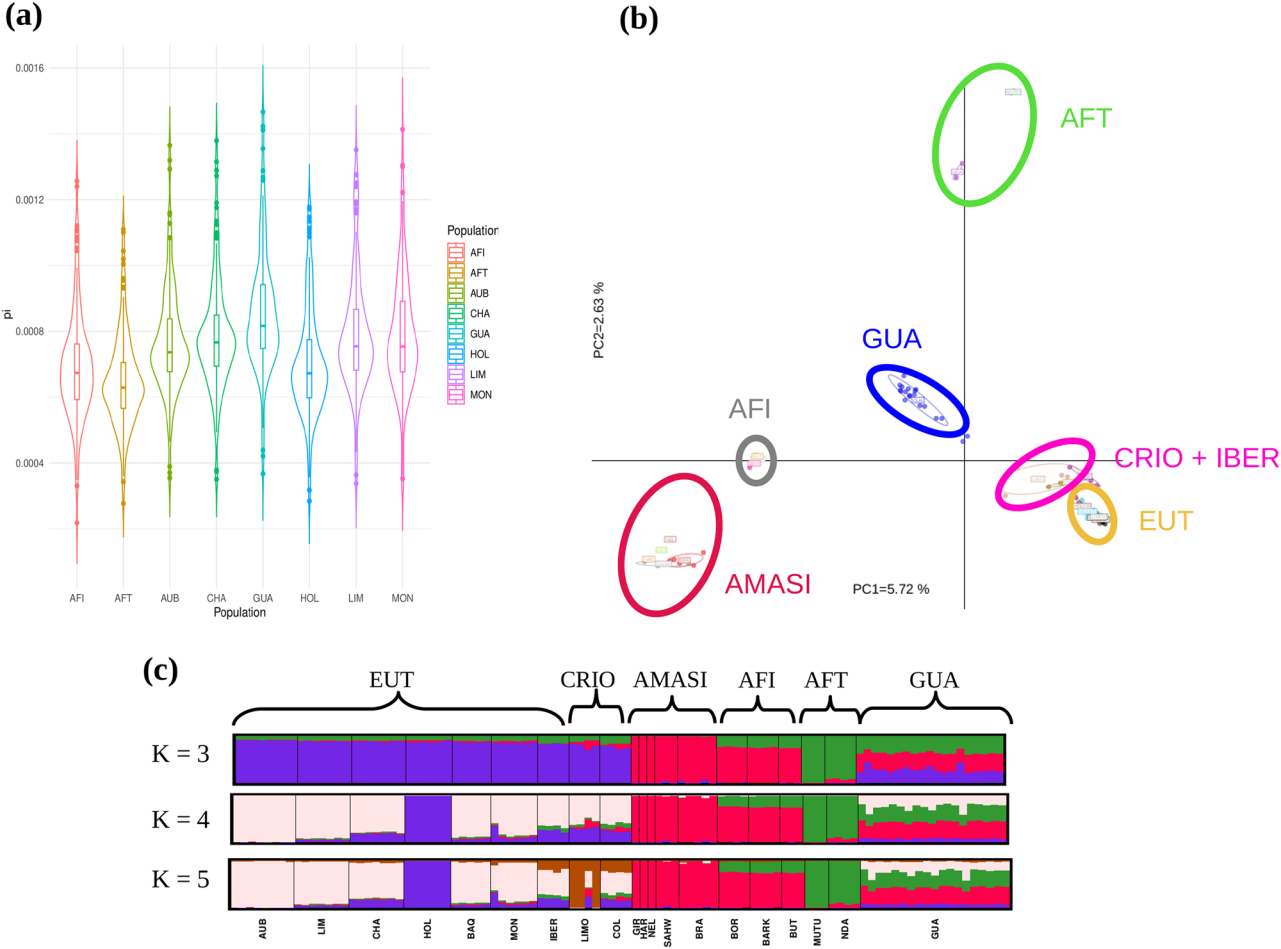
Genetic diversity and population structure and genetic diversity of Creole cattle from Guadeloupe (GUA). Population names and locations are described in supplementary table S13 (supplementary material). (**a**) Genome-wide distribution of nucleotide diversity (Pi) estimated in 1-Mb nonoverlapping window. *AFI* African indicine, *AFT* African taurine (**b**) Principal component analysis, PC 1 against PC 2. *CRIO* Criollo (Colombian and venezuelan cattle breeds), *IBER* Spanish breeds, *AMASI* American-Australian and Asian indicine breeds (**c**) Results of admixture analysis for K 3–5.

To gauge the level of within-population genetic diversity, we computed nucleotide diversity in windows of 1 Mb across the cattle genome. Nucleotide diversity is defined as the average number of nucleotide differences per site between two randomly chosen DNA sequences in a population^14^. The Creole cattle from Guadeloupe population has the highest nucleotide diversity (median = 8.16 × 10^−4^) which is consistent with their three-way admixture. African taurine has the lowest diversity (median = 6.28 × 10^−4^) (Fig. 1a) which can mainly be explained by a higher level of inbreeding. This is reflected by both a higher number of ROH and a larger cumulative ROH length compared to the other populations. Conversely, ROH detection in windows of minimum amount of 100 homozygous SNPs (see “Methods” section) revealed that GUA has the lowest number of ROH (average number of 153 ± 34 ROHs) (Supplementary Fig. S1a). Additionally, together with African indicine populations, GUA has most of its individuals with a total ROH length per individual, below 200 Mb (Supplementary Fig. S1b).

A total of 12 ROH islands located on chromosomes 1, 5, 6, 7, 10, 11, 12 and 19 were identified in the GUA genome. Among the detected ROH islands, the strongest pattern was observed on BTA11 (34,764,780–34,919,599 bp), BTA05 (48,438,356–49,020,572 bp) and BTA12 (28,635,496–28,916,516 bp) with an overlapping ROH region present in 47%, 41% and 41% of the samples, respectively (Supplementary Table S4).

### Population structure and genetic relationship analyses

Principle component analysis (PCA) grouped individuals in clusters according to their populations of origin (Fig. 1b). The first principal component (PC1) explains approximately 5.72% of the global variation and formed a gradient between American–Australian and Asian indicine cattle on one hand and European taurine on the other hand. The second principal component (PC2) explained approximately 2.63% of the global variation and defines the differences between African and European taurine. The three African indicine (AFI) populations, Barka (BARK), Boran (BOR) and Butana (BUT) are in the same genetic cluster and maintain a certain distance from the American–Australian and Asian Indicine (AMASI) group. GUA fell at an intermediate position between AFI and EUT groups. It is worth noting that the five Criollo (CRIO) breeds (the Colombian and Limonero individuals) are very close to the Iberian group with the latter being very close to the other European breeds. *ADMIXTURE* analysis also recapitulated these findings (Fig. 1c). When K = 3, European breeds (blue) were separated from American–Australian and Asian indicine (red) and African taurine (green). GUA individuals have, on average, 29%, 35% and 36% of EUT, AFT and IND ancestries, respectively. When K was set to 4, the European ancestry of GUA is mainly from southern Europe while the Criollo cattle have a higher proportion of Northern European ancestry [represented by Holstein (HOL)] and very little proportion of AFT and IND ancestries. Increasing K to 5, separates Limonero (LIMO) from the Colombian breeds. From K = 3 through K = 5, the three African zebu populations showed a similar genetic structure composed exclusively of indicine (75% on average) and African taurine (25% on average) ancestries. We ran the ELAI algorithm to infer AFT, EUT and indicine ancestries in each GUA individual. The results show that the contributions of each of the three ancestries are not homogeneous across individuals. For instance, individuals GUA14 and GUA2 have the highest proportions of EUT introgression (42.2% and 41.6%, respectively). This contrasts with individuals GUA15 and GUA16 which have only 16% of EUT ancestry (Supplementary Fig. S2). Likewise, the proportions of the three ancestries vary widely across chromosomes. For instance, AFT ancestry in GUA8 [individual with the highest global AFT ancestry (48.7%)] varied between 36.2% on the BTA04 and 63.3% on the BTA10 (Supplementary Fig. S2).

Pairwise Fst values obtained with Genepop corroborated the genetic proximity within AFI populations (0.016 < Fst < 0.044) and between these latter and GUA (0.049 < Fst < 0.062) while Fst estimates between GUA and AMASI breeds were almost two times greater (Fst > 0.1). Moderate values of Fst are observed between GUA and EUT breeds with a South European origin [the Spanish breeds (IBER), Blonde d’Aquitaine (BAQ), Limousin (LIM) and Charolais (CHA)] (0.0691 < Fst < 0.0799) (Supplementary Table S5).

We have examined our data with the TreeMix software which allows for modelling both population splits and gene flow between populations. Ten migration events were sequentially added to the phylogenetic tree which explained 99.79% of the model’s variance of relatedness between populations. The resulting phylogenetic network structure shows GUA as a sister population to Ndama. Both populations are in clade with the East African Zebu, Butana. The other two African zebu populations (BOR and BARK) were in clade with the Asian zebu (BRA and SAHW). TreeMix shows GUA strongly introgressed with the African taurine Muturu (MUTU) and the East African zebu, Barka. A third edge originating more basally in the phylogenetic network was also placed towards GUA. High levels of admixture are shown between African taurine (MUTU and Ndama) and African zebu (BOR and BUT) (Supplementary Fig. S3).

### Selection signature detection in Creole cattle from Guadeloupe

**Figure 2:**
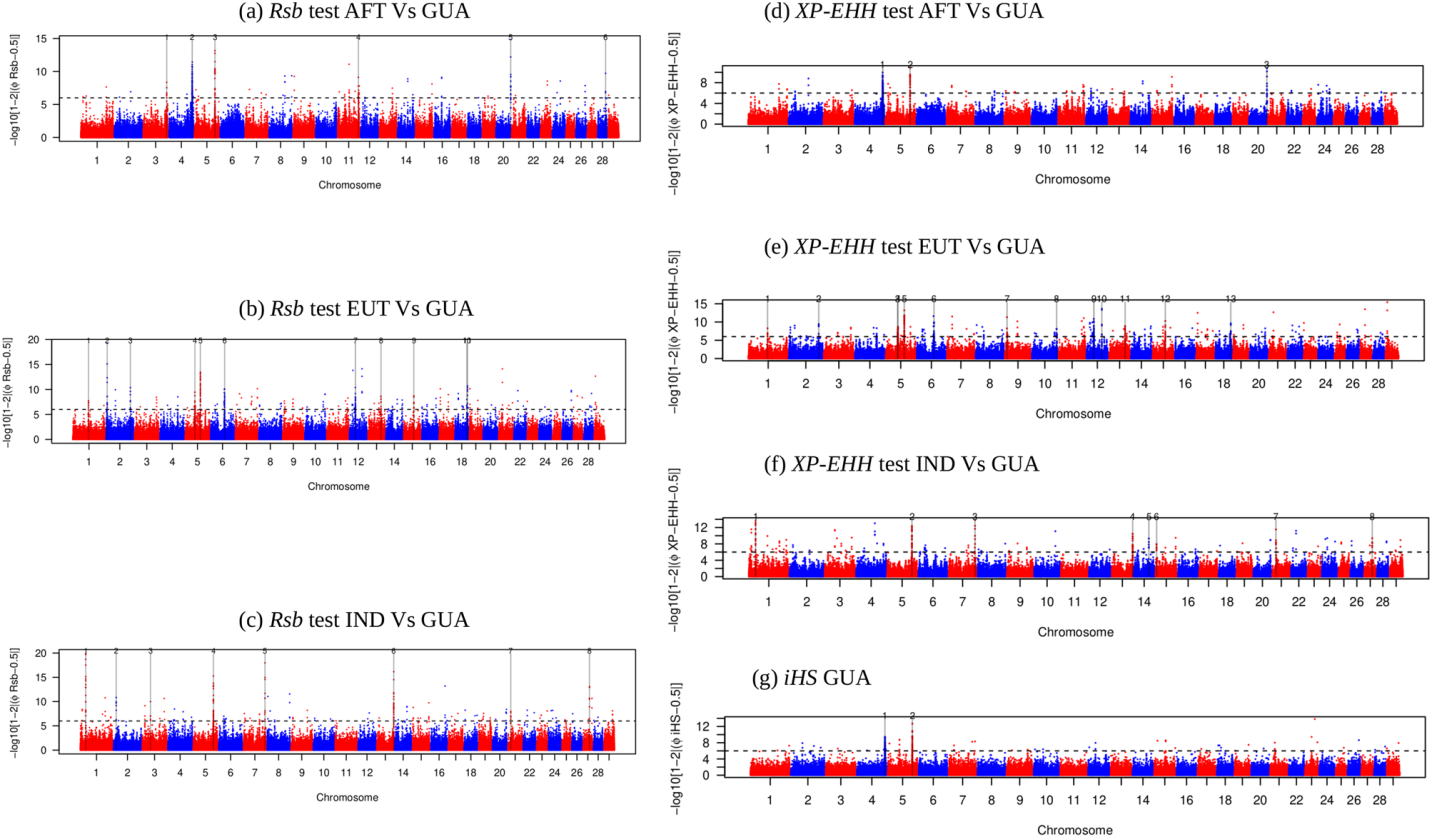
Manhattan plots showing the results of Extended Haplotype Homozygisty-based tests. (**a**) *Rsb* test AFT versus GUA cattle. (**b**) Rsb test EUT versus GUA cattle (**c**) Rsb test IND versus GUA cattle. (**d**) *XP-EHH* test AFT versus GUA cattle. (**e**) *XP-EHH* test EUT versus GUA cattle (**f**) *XP-EHH* test IND versus GUA cattle. (**g**) *iHS* test for GUA cattle. Horizontal dashed lines mark the significance threshold applied to detect the outlier SNPs (− log10 (*p* value) = 6).

**Figure 3:**
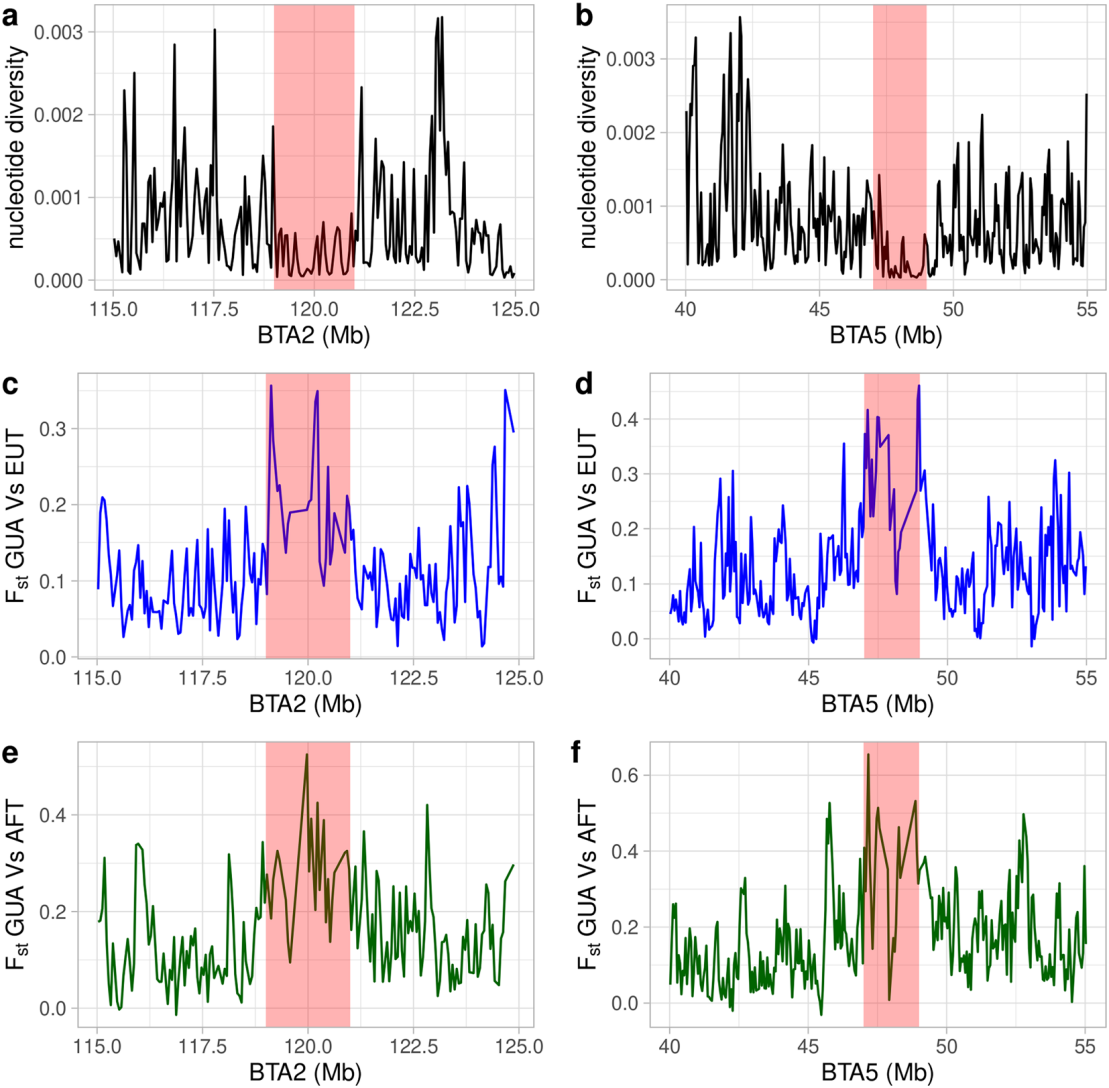
Nucleotide diversity and Pairwise Fst values [EUT vs. GUA (blue) and AFT vs. GUA (green)] calculated for each 50-kb window around the candidate regions on chromosomes 2 and 5. (**a**) Nucleotide diversity on chromosome 2. (**b**) Nucleotide diversity on chromosome 5. (**c**) Pairwise Fst values between GUA and EUT on chromosome 2. (**d**) Pairwise Fst values between GUA and EUT on chromosome 5. (**e**) Pairwise Fst values between GUA and AFT on chromosome 2. (**f**) Pairwise Fst values between GUA and AFT on chromosome 5.

To detect genomic regions putatively contributing to local adaptation in GUA cattle, we used three EHH-derived statistics (*iHS*, *Rsb* and *XP-EHH*) based on the decay of haplotype homozygosity as a function of recombination distance. Candidate regions were defined by groups of at least five outlier SNPs exceeding the significance threshold of − log10 (*p* value) = 6. The rationale is that selective sweeps tend to produce clusters of extreme scores across the sweep region, while under a neutral model, extreme scores are scattered more uniformly^15^. *Rsb* and Cross-population Extended Haplotype Homozygosity (*XP-EHH*) statistics were computed at each SNP. Haplotypes estimated in each population were pooled, for each autosome, according to their group of origin (AFT, EUT and IND). In total, 14, 102 and 44 haplotypes were thus considered representative of AFT, EUT and IND ancestries, respectively. *Rsb* detected 6, 11 and 8 regions putatively under selection for GUA/AFT, GUA/EUT and GUA/IND comparisons, respectively (Fig. 2a–c and Supplementary Table S6). *XP-EHH* identified 3, 13 and 8 regions putatively under selection for GUA/AFT, GUA/EUT and GUA/IND comparisons, respectively (Fig. 2d–f and Supplementary Table S6). The two significant windows on BTA04 (at position: 113–113.5 Mb) and BTA05 (at position: 99–99.5 Mb) revealed by the intra-population *iHS* test (Fig. 2g) were among the candidate regions jointly detected by *Rsb* and *XP-EHH* tests (Supplementary Table S6). The variant under strongest selection on chromosome 4 (− log(*p* value) > 11) fell within GIMAP genes. On chromosome 5 the highest signal fell 16 Kb upstream NKG2-A/NKG2-B type II integral membrane protein. Overall, 17 candidate regions were identified by at least two EHH-based tests (Supplementary Table S6) of which six regions have significantly positive *Rsb* values suggesting that they are under selection in GUA. Of these, five regions located on BTA02 (at position: 120–120.5 Mb), BTA05 (at position: 47–47.5 Mb), BTA06 (at position: 69–69.5 Mb), BTA12 (at position: 29–30 Mb) and BTA13 (at position: 63.5–64 Mb) were identified in the EUT versus GUA comparison. The first four candidate regions overlapped with areas harbouring a sudden increase in indicine allele dosage (Supplementary Fig. S4). Importantly, these four regions are also among the top 1% regions with the highest indicine ancestry in the whole genome. The fifth candidate region (located on BTA13 at position: 63.5–64 Mb) did not show in itself a high indicine ancestry. Rather, an excess of zebu ancestry is observed in its immediate vicinity (position: 65.175–65.775 Mb) (Supplementary Table S7). It is worthy to note that all five aforementioned regions showed a reduced level of genetic diversity within GUA and an increased level of genetic differentiation between GUA on the one hand and AFT and EUT on the other hand (Fig. 3 and Supplementary Fig. S5). Furthermore, these five regions overlapped with one of the top 1% windows showing the highest genetic differentiation with EUT and AFT breeds (Supplementary Tables S8 and S9).

We also tested for signals of selection using the composite likelihood ratio implemented in SweeD software to detect patterns of site frequency spectrum in the GUA population. We found that all five candidate regions had the highest CLR values in their respective chromosomes (Supplementary Fig. S6). Genes that fell within the peak area of the aforementioned five candidate regions in addition to the outlier window on the BTA04 (at position 113–113.5 Mb) detected with the *iHS* test were considered as relevant candidates (Table 1). On chromosome 2, the highest signal is 73.5 Kb downstream of *EIF4E2* gene. On chromosome 5, the two variants with the highest *p* value fell within *GRIP1*, a gene that facilitates the anti-inflammatory effects of glucocorticoids in vivo^16^. We identified another gene in this region: *DNA Helicase B (HELB)* located 250 Kb upstream the highest signal. In candidate region on chromosome 12, the three SNPs of highest significance (8.48 < − log(*p* value) < 10.4) are less than 80 Kb upstream of relaxin family peptide receptor 2 (*RXFP2*). On chromosome 13, the cluster of the four most significant outlier SNPs is less than 100 Kb upstream the *ASIP* gene.

**Table 1:** The most relevant candidate genes putatively under selection in the Creole cattle from Guadeloupe.

BTA	Region (Mb)	Gene closest to the most significant SNPs	Phenotype	References
4	113–113.5	*GIMAP4, GIMAP5, GIMAP7*	Parasite resistance	^66^
2	120–120.5	*EIF4E2*	Response to exercise	^46^
5	47–47.5	*HELB*	Response to DNA damage, reproductive traits, yearling weight	^36^
		*GRIP1*	Immune response (anti-inflammatory actions of glucocorticoids)	^16^
6	69–69.5	*LNX1*	Neuronal signaling and anxiety-related phenotypes	^67^
12	29–30	*RXFP2*	Horn size	^40^
			Thermoregulation	^43^
13	63.5–64	*ASIP*	Coat color	^68^

## Discussion

### A unique population structure of GUA

In this paper, we present a characterization of the first complete genome sequence of the Creole cattle from Guadeloupe. In line with previous reports^7^, our results highlight the three-way, European taurine x African taurine x indicine admixture in the Creole cattle from Guadeloupe. Quantifying the amount of each of the three ancestries across the chromosomes indicate that, despite some variation observed in admixture proportions between GUA individuals’ genomes, there is a clear trend in favour of a dominance of non-European ancestries in the genome of almost all GUA animals. Conversely, we found high proportions of EUT ancestry in the other Creole breeds (Fig. 1c) leading to a clear separation between these and GUA in our data set regardless of the analytical method we used (Fig. 1b,c and Supplementary Fig. S3). More particularly, the GUA population is not as much as closely related to Iberian cattle as the other Criollo breeds used in the present study do (Fig. 1b,c). Previous studies found that American Criollo cattle originate from Iberia with African ancestry inherited via Iberian ancestors^17^. Our results suggest that this does not seem to be the case of the Creole cattle from Guadeloupe breed. The various analyses are consistent with a model wherein the GUA population originates from a direct introduction of African taurine cattle to Guadeloupe^7,18^. A previous analysis of sequence variation in the hypervariable segment of the mitochondrial DNA control region (mtDNA) similarly reported a high proportion of African mtDNA haplotypes in twenty-five GUA individuals^4^. Interestingly, the aforementioned study included Creole samples from Antigua and St. Lucia, two Guadeloupe’s neighbouring regions and found that mtDNA pools of these samples are predominantly European, with very low African mtDNA admixture proportions. Moreover, when using microsatellite data, the authors of the study reported that the Guadeloupe cattle was the only population to exhibit West African–specific alleles^4^. This further confirms the uniqueness of this population with respect to the other Creole breeds even those found in neighbouring regions and which are expected to have a similar genetic history.

The lower genetic differentiation and a closer position in PCA analysis between GUA and East African zebu compared to Indian zebu and the migration edge placed by TreeMix between GUA and the East African Zebu Barka, all suggest that the indicine ancestry in the GUA genome has an African origin. African indicine ancestry in the GUA genome could be inherited via west African taurine which also carries an indicine component in their genome stemming from past admixture events between migrating populations of East African zebu and local taurine cattle in West Africa. This admixture was previously reported^19^ and is recognizable in our phylogenetic network where migration edges are placed between African taurine (MUTU and Ndama) and African zebu (BOR and BUT) (Supplementary Fig. S3). Another plausible hypothesis is that African indicine ancestry of the GUA cattle could be inherited from African zebu populations accompanying nomadic people such as Fulani. Pastoral populations represent a major force for genetic exchange between taurine and indicine cattle all over central and West Africa through transhumance of their livestock along the African Sahel^12^. Written records suggest that nomadic herders spread from West Africa (currently Senegal, Guinea, Mauritania) around 1000 years ago, reaching the Lake Chad Basin 500 years later^20^. Clearly, estimating with high accuracy the origin of African indicine ancestry in the GUA genome would require using additional cattle populations from western Africa and the Sahel region.

### Detecting selection signature in the GUA genome

To identify footprints of selection in the genome of GUA cattle, we used pools of individuals haplotypes belonging to contemporary populations sampled from geographical locations remote from each other, as proxies for their assumed ancestral counterparts. In this regard, EUT ancestry was represented by breeds from France, Spain as well as Criollo populations which were shown to be genetically close to the former breeds (see Fig. 1b,c (K = 4)). Likewise, indicine ancestry was represented by zebu breeds from East Africa, America, Australia and the Indian subcontinent. Such a haplotype pooling applied to an extensive comparison of groups of populations would ‘smooth out’ effects specific to each of the populations from a given ancestry^7^.

As a first step, we applied two types of complementary EHH-based statistics, the integrated Haplotype Homozygosity Score (*iHS*) reflecting primarily ongoing selection, *Rsb* and *XP-EHH*, having the most power to detect completed selection after fixation of the advantageous allele^15,21,22^.

Two candidate regions on chromosomes 4 (position: 113–113.5 Mb) and 5 (position: 99–99.5 Mb) were jointly identified by the two types of approaches. Additionally, we observe that the candidate region on chromosome 4 is also under selection in AFT since it has a negative *Rsb* value. This is further supported by the identification of this region when we computed the *Rsb* statistic for AFT versus EUT and AFT versus IND comparisons (Supplementary Tables S10 and S11). The strongest selection signal in this region fell within *GIMAP* genes which play a central role in lymphocyte maturation and lymphocyte-associated diseases^23^. *GIMAP5* knockout mice have complete loss of natural killer cells^24^ which are critical to the protective response during *Trypanosoma cruzi* infection^25^ widespread in West Africa. Taken together, these findings lead us to speculate *GIMAP* genes are under ongoing selective pressure in the GUA genome owing to the presence of novel pathogens in the Caribbean islands (compared to those present in Africa). Infectious pathogens are among the strongest selective forces that shape the genome of several mammalian species such as human^26^.

One of the main drawbacks of selection signature detection methods is their elevated rate of false positives^27^. Limiting the number of spurious signals that can arise owing to various confounding factors such as the marker discovery process and/or population demographics is the main challenge in genome-wide scans aiming at the detection of selective sweeps. In the present study every attempt has been made to reduce the number of false-positive signals while focusing on candidate regions under selection in the GUA genome. First, the use of whole genome sequencing should reduce SNP ascertainment bias afflicting commercial genotyping arrays. Avoiding SNP ascertainment bias is critical for accurate population genetic analyses because levels of variability, distribution of allele frequencies, and levels of linkage disequilibrium will all be strongly affected by such ascertainment schemes^28,29^. Second, in our EHH-based tests, we relied on consistency of signals over regions, that is taking clustering of highly significative outliers as evidence for selection. Third, and most importantly, we considered that selection signals that are consistently supported across different statistical tests are less likely to be false-positives. Indeed, although these tests are designed to detect selective sweeps that vary in terms of type, age and strength of selection events, selection signals supported across different methodologies can increase power, reduce sensitivity to confounding factors (which are unlikely to affect different methods in a similar manner) and increase precision of the detection of the selective sweep^30^. Here, we used various methods based on excess of haplotype homozygosity, deformation of the allele frequency spectrum, excess of differentiation in allelic frequencies between the GUA population and proxies of its ancestral populations and reduction in genetic diversity around the selected region to identify reliable selection signatures in the genome of GUA. Fourth, we relied on the identification of excess/deficiency of local ancestry in the GUA genome to further confirm selection signals revealed by the various tests we used. The rationale is that, under a recent admixture scenario followed by a strong selection, we expect a parallel increase in local ancestry proportions in the regions surrounding the beneficial variants. We found evidence of congruent signals between methods for five candidate regions on chromosomes 2, 5, 6, 12 and 13, all of them identified in the GUA versus EUT comparison. These five regions exhibit a sudden increase in indicine ancestry (Supplementary Fig. S4), clusters of highly significant SNPs in *Rsb* and *XP-EHH* tests (Fig. 2), low nucleotide diversity and high differentiation levels between GUA on the one hand and EUT and AFT on the other hand (Fig. 3 and Supplementary Tables S8 and S9). These regions also displayed the highest CLR values on their respective chromosomes (Supplementary Fig. S6). A further result is that the two regions on chromosomes 5 and 12 overlap with ROH islands including at least 35% of the individuals (Supplementary Table S4). Altogether these findings validate the five genomic regions as the most biologically relevant results and support previous studies suggesting that adaptation of the GUA cattle to tropical environment occurred mainly through its indicine ancestry^7^.

Adopting stringent criteria to declare candidate regions in our EHH-based tests (5 SNPs exceeding the significance threshold of 10^−6^ within 500-Kb windows (see “Methods” section) constitutes a potential limitation of our study since such approach is likely to lead to a large number of missed selection signals. This might partially explain the little congruence with the candidate genomic regions reported by Gautier and Naves 2011. When we remade *Rsb* calculation for EUT versus GUA comparison using the same criteria reported by these authors (threshold *p* value = 10^−4^ in 1-Mb sliding windows with 500-kb overlapping step and one marker exceeding the significance threshold by window), we detected 278 regions of which 13 reported in Gautier and Naves 2011 (out of 16 candidate regions) (Supplementary Table S12).

### Biological function of the most relevant candidate genes located within the relevant candidate regions

The highest selection signals in the five strong candidate regions showing an excess of indicine ancestry fell within or nearby genes involved in stress response to tropical constraints and probably to some human-driven socio-cultural pressure (Table 1). Many of our candidate genes have been reported in other species. For instance, *ASIP,* a gene linked to skin pigmentation in human^31^ and mice^32^ was also localized in a strong selective sweep in Indian water buffalo^33^. *ASIP* was previously reported to be associated with darkness of hair coat in Nellore cattle^34^. Coat colour is an important potential adaptive function that helps regulate body temperature in mammals^35^.

Our results demonstrate that the Creole cattle from Guadeloupe which are usually exposed to long periods of direct, intense sunlight either during grazing or during ploughing, possess signatures of putative selection within or around genes associated with thermo-tolerance. Aside from *ASIP* gene, the strongest candidates within the region on chromosome 5 are *GRIP1* and *HELB.* Both genes were previously identified in a 430-kb selective sweep in Asian indicine cattle^36^. Importantly, *HELB* is known to be involved in the response to DNA damage and replication stress^37^ that could be induced by prolonged exposure to solar ultraviolet radiation^38^. We also confirmed the adaptive role of *Relaxin family peptide receptor 2* (*RXFP2*) gene, previously identified by Gautier & Naves 2011. *RXFP2* is a gene with a pleiotropic effect. It affects both inguinoscrotal testis descent^39^ and horn size in wild bighorn^40^ and domestic sheep^41,42^. The use of horns is likely part of a thermoregulatory mechanism in several pecoran species. Indeed, since the core of the horn is part of the sinus, horns may contribute to nasal heat exchange, a mechanism that considerably reduces water loss through cooling of the air during exhalation^43^. Another explanation to the selection pressure exerted on *RXFP2* is that Creole bulls were traditionally used for sugarcane cart pulling and cattle cart race competition, with a yoke attached to the horns. This has probably led to the development of a stronger horn base^7^. Creole cattle have been historically selected for draught works in sugarcane plantation. They are known to have a better endurance in long-term effort than crossbreed or exotic breeds^44^. Accordingly, we found that the strongest evidence of selective pressure on chromosomes 2 co-localizes with *EIF4E2.* In human cells under hypoxia, eIF4E2 plays a fundamental role in protein synthesis. This gene substitutes its homologue, eIF4E and forms a complex with the oxygen-regulated hypoxia-inducible factor 2α^45^. *EIF4E2* was among the candidate genes that were shown to be under positive selection in Fu Zhong buffalo characterized by strong muscles and able to endure the strength to pull a plough through muddy rice paddies^46^. We also identified another gene, *OCIAD1,* as a good candidate for mitochondrial adaptation during exercise. *OCIAD1* lies within a 3-Mb region which is among the top 1% regions with the highest indicine ancestry and is located in the vicinity of our candidate region on BTA06. The region encompassing this gene is detected by *Rsb* and *XP-EHH* tests when relaxing the *p* value threshold to 10^−5^. Almost ~ 11% of the 220 SNPs contained in this region (66.5–68 Mb) exceed the significance threshold (data not shown). Importantly, the two variants with the highest *p* value in this region are located 16 Kb upstream *OCIAD1* which encodes a mitochondrial inner membrane protein that regulates mitochondrial Complex III assembly in cells^47^. The latter is among the complexes that play a key role in electron transport and proton gradient production, precisely across the inner mitochondrial membrane^48^. Proton gradient provides the energy necessary for the production of ATP whose demand increases in the muscle with exercise intensity^49^. Selection for draught traits in the GUA population thus seems to have provoked responses in a diversity of pathways involving at least *EIF4E2, OCIAD1* and *RXFP2* genes. Such selective pressure promoted the fixation of beneficial alleles from an indicine origin that allowed GUA individuals to cope with withstanding hours of high intensities of physical activity.

In this study, we have generated for the first time a catalogue of genetic variants found in the Creole cattle from Guadeloupe. We were able to show that GUA adaptation to local environment occurred mainly through its indicine component. We also demonstrate that pathogenic environment, thermotolerance and physical stamina are important drivers of local adaptation in Creole cattle. Overall, our results provide clues for understanding the adaptive admixture in the Creole cattle from Guadeloupe thus contributing to the emerging picture of the genes and pathways associated with traits resilience in livestock species. Our study may represent a starting point for a targeted and sustainable genetic breeding improvement of Creole cattle.

## Methods

### Ethics declaration

Blood collection was done according to good practices recommended for identification of sires for paternity checking in France. Semen was collected for the main purpose of insemination, according to relevant technical guidelines for semen collection and preparation. This study was approved by the scientific committee of the Metaprogramme SELGEN of INRA, which afforded a grant to the project TROCADERO. The study is in accordance with ARRIVE guidelines.

### Sample selection and genome sequencing

Twenty-three Creole bulls representative of the INRA nucleus in Guadeloupe were selected for the purpose of this study. INRA experimental nucleus was created in 1980 from local animals chosen according to their phenotype. New local sires are regularly introduced in the nucleus as a part of a conservation program aiming to avoid loss of variation in the GUA population. Ten out of the twenty-three individuals of the present study are sons of founder sires of the experimental flock while the remaining thirteen animals are unrelated sires (based on genealogical records and sampling sites) chosen from the local stock of Guadeloupe before introduction to the INRA nucleus. The animals selected for the purpose of this study are among the most frequently used in artificial insemination.

Genomic DNA was extracted from whole-blood and semen samples collected between 1995 and 2015. Four Colombian individuals belonging to the IMAGE project (H2020 project 677353 'Innovative Management of Animal GEnetic resources) were also included in the study. Paired-end libraries with insert size of 500 bp were constructed for each individual and sequenced using the HiSeq 3000 platform (Illumina) in the Genome et Transcriptome (GeT) GénoToul platform (Toulouse, France), following the manufacturer’s protocol.

### Sequence alignment and genotype calling

Genotype data were generated following the 1000 Bull Genomes Project Run 8 guideline^50^. Quality control of raw sequence reads was performed using the fastQC software v.0.11.7 (http://www.bioinformatics.bbsrc.ac.uk/projects/fastqc/). Trimmomatic-0.36^51^ was used to remove Illumina adapter sequences, low-quality bases and artefact sequences. Filtered sequences were then mapped against the bovine reference genome (ARS-UCD1.2) using the Burrows-Wheeler Alignment tool (bwa mem v.0.7.17)^52^ with default parameters. The resulting SAM files were then converted to BAM format, sorted, and indexed using SAMtools^53^. Potential PCR duplicates were removed using the MarkDuplicates tool from Picard version 1.88 (http://broadinstitute.github.io/picard). Only properly reads with a mapping quality of at least 30 were kept. Prior to variant discovery, local realignment was performed using two GATK (Genome Analysis Toolkit) version-3.8-1-0-gf15c1c3ef modules, RealignerTargetCreator and IndelRealigner. GVCF files were then created for each sample using the GATK HaplotypeCaller module. Single nucleotide polymorphisms (SNPs) and small insertions and deletions (indels) were subsequently called for all samples using the GATK GenotypeGVCFs tool. VCFtools v.0.1.17 software was used to filter out indels, multiallelic SNPs and variants that (1) displayed a minor allele frequency (MAF) < 0.1; (2) have missing genotype rates > 0.1; (3) did not pass Hardy–Weinberg equilibrium (HWE) test (*p* < 0.01) and (4) have a quality score (– minQ option) below 100. The selected variants were subsequently annotated using the SnpEff annotation software^54^.

### Data merging and relatedness check

We downloaded publicly available genomes of of 41 individuals including 12 AFI, 10 AFT, 7 Australian-American indicine (AMI), 4 Asian indicine (ASI), 4 European taurine breeds from Spain (IBER) and 4 LIMO individuals (representative of Criollo (CRIO) cattle) (Supplementary Table S13). We followed the same procedure aforementioned in sequence alignment and variant calling. Finally, we included in the data set, genotyping data belonging to 50 individuals from six French breeds provided by the 1000 Bull Genomes Project (Supplementary Table S13). We applied the same marker selection thresholds (maf = 0.1, missing genotype rate = 0.1, *p* value HWE = 0.01 and minQ = 100) to build these two databases which resulted in 16,360,962 and 8,955,346 high-quality SNPs for the public and the 1000 Bull Genomes genotyping data, respectively. All three databases were combined into a single one containing 5,316,956 common snps. Nucleotide diversity (Π) at a whole-genome scale was estimated in sliding windows of 1 Mb using VCFtools considering the following breed groupings: AFI (N = 11) and AFT (N = 7) and the each of the following breeds separately: Aubrac (AUB; N = 8), Charolais (CHA; N = 7), Holstein (HOL; N = 6), Limousin (LIM; N = 7), Montbeliarde (MON; N = 6). In Π computation, we selected 10 GUA individuals among the least related. This was done to have sample groups with similar size (for a matter of comparison).

We further performed an LD-based SNP pruning using PLINK^55^ with the “-indep-pairwise 20 4 0.6” option which resulted in 1,064,342 SNPs spread over all autosomal chromosomes used in population structure analyses. Average inter-marked distance was 2.3 Kb ± 4.3 Kb (Supplementary Table S14). To overcome the effect of closely related animals, we performed a relatedness test between individuals within each population using PLINK. The software calculates a variable called PI-HAT reflecting extended haplotypes shared between distantly related individuals. We excluded one individual from each pair of individuals with a PI-HAT value > 0.05 which is a value roughly corresponding to 3rd-degree relationships. In total, after relatedness filtering, 99 individuals including 19 GUA animals, were available for the different analyses (Supplementary Table S13).

### Population structure and genetic relationship analyses

We performed a principle component analysis (PCA) using the adegenet R package^56^. Furthermore, genetic structure was inferred from SNP data in ADMIXTURE 1.3 software^57^. We ran ADMIXTURE for values of K from 3 through 5. DISTRUCT software^58^ was used to graphically display ancestry within each individual. Global averages of pairwise population differentiation measured by fixation index (*Fst*) was estimated using Genepop 4.6 software^59^ for populations represented by more than one animal. The four Spanish individuals were considered as one population as well as the four Colombian animals. Next, we performed local ancestry assignment across the GUA genome using the Efficient Local Ancestry Inference (ELAI) algorithm^60^ under a three-way admixture model using AMASI and AFI populations as proxies for indicine ancestry, Muturu and Ndama as proxies for AFT ancestry, the French and the Spanish breeds as proxies for EUT ancestry. ELAI uses a two-layer hidden Markov model to detect the structure of haplotypes for unrelated individuals. The algorithm models two scales of linkage disequilibrium (one within a group of haplotypes and one between groups) and provides a map, for each admixed individual, showing the probability, for each SNP, to descend from each one of the ancestral populations. ELAI analysis was conducted across 19 GUA genomes by setting the parameters -mg (number of generations) to 70, -s (EM steps) to 30, -C (upper clusters) to 3, and -c (lower clusters) to 15.

The patterns of population splits and mixtures were inferred using TreeMix^61^. To run TreeMix, we considered the 17 populations with more than two individuals. We built a maximum likelihood tree using blocks of 30,000 SNPs with no migration events allowed. Then, we built a phylogenetic tree of these populations and started adding migration events (modelled as edges) sequentially to the phylogenetic model. The migration edges were added until 99.79% of the variance in ancestry between populations was explained by the model.

### Identification of runs of homozygosity

Runs of homozygosity (ROHs) were identified in sliding windows of 100 SNPs using PLINK and the 1,064,342 SNPs that passed quality control filtering. ROH were detected within the following five breed groupings: Creole cattle from Guadeloupe (GUA), African Taurine (AFT) : Muturu and Ndama, Africain Indicine (AFI) : BOR, BUT and BARK, European Taurine (EUT) : IBER (represented by the four Spanish breeds), AUB, CHA, BAQ, LIM, MON, HOL, LIMO, COL (represented by the four Colombian breeds) and American-Australian and Asian Indicine (AMASI) : BRA, GIR, NEL, HAR and SAHW. The following parameters were used to define a ROH: (1) homozyg-snp (minimum number of SNPs that a ROH is required to have): 100. (2) homozyg-density (required minimum density to consider a ROH): 50. (3) homozyg-gap (length in Kb between two SNPs in order to be considered in two different segments): 1000 (4) homozyg-window-het (number of heterozygous SNP allowed in a window): 3 (5) homozyg-window-missing (number of missing calls allowed in a window): 5. The –homozyg-group option implemented in PLINK was used to assess ROH islands shared among GUA individuals. These were defined as the homozygous segments shared by at least 30% of the samples.

### Identification of selection signatures

Prior to selection signature analysis, we performed a more stringent LD-based pruning with the ‘-indep-pairwise 20 4 0.4’ option of PLINK which resulted in 433,408 SNPs. Integrated haplotype score (*iHS*)^15^, *Rsb*^22^ and cross population extended haplotype homozygosity (*XP-EHH*)^21^ scans were performed using the *rehh* package^62^. In *iHS* computation, the information on the ancestral and derived allele state is needed for each SNP because this statistic is based on the ratio of the extended haplotype homozygosity (EHH) associated to each allele. In our analysis, the ancestral allele was inferred as the most common allele within our dataset. *iHS* scores for each SNP were transformed into two-sided *p* values: p*iHS* = − log10[1 − 2|Φ(*iHS*) − 0.5|]. *Rsb* and *XP-EHH* analyses were performed for each of the three pairwise comparisons: GUA versus AFT, GUA versus EUT (French, Spanish and Criollo breeds) and GUA versus IND (AMASI and AFI populations). Haplotype phasing was performed using fastPHASE 1.4^63^. Since fastPHASE is based on haplotype clusters, whose size should be set a priori, we used the toolkit implemented in imputeqc R package^64^ to estimate the optimal number of haplotype clusters (K) needed for haplotype phasing. Imputeqc package has been designed to assess the imputation quality and/or to choose the model parameters for imputation. In the present study, we found that K = 20 provided the best imputation quality (for 5% of masked data). Therefore, we used this value to run fastPHASE. Considering that *Rsb* and *XP-EHH* values are normally distributed, a Z-test was applied to identify significant SNPs under selection. Two-sided *p* values were derived as p*Rsb* = − log10[1 − 2|Φ(*Rsb*) − 0.5|] and p*XP-EHH* = − log10[1 − 2|Φ(*XP-EHH*) − 0.5|] where Φ (x) represents the Gaussian cumulative distribution function. For all three EHH-based tests, the maximum allowed gap between two SNPs was set to 500 Kb. We used sliding nonoverlapping 500-Kb windows to perform selection signature detection. A window is classified as putatively under selection when it contains at least 5 markers exceeding the significance threshold of − log10 (*p* value) = 6. Positive *XP-EHH* and *Rsb* values indicate longer haplotypes in the target population (i.e. GUA) therefore suggesting that selection occurred in the GUA population.

### Confirming the relevant candidate regions putatively under selection in GUA

Regions putatively under selection in the GUA genome, identified by at least two EHH-based tests and experiencing a sudden increase in one of the three ancestries of GUA compared to the average level on their respective chromosomes were considered as relevant. We employed additional approaches to further confirm these relevant candidate regions. First, Fst and nucleotide diversity were calculated for nonoverlapping 50-Kb windows across the genome using VCFtools. Highly differentiated windows with at least 4 SNPs between GUA and each of its three ancestries were identified. We checked if our candidate regions were among the top 1% windows and if these regions present specific patterns of nucleotide diversity compared to the neighboring regions. Next, we used SweeD v4.0.0 software^65^ to calculate the composite likelihood ratio (CLR) in nonoverlapping 500-kb windows along the chromosomes containing the relevant regions. The software detects Site Frequency Spectrum (SFS) patterns generated by complete selective sweeps.

We collected information through a literature search to discuss the biological implications of our findings with regard to the candidate genes located in the relevant genomic regions known to be involved in phenotypic variation of adaptive traits.

## Supplementary Information

Supplementary Information.

## Data Availability

The generated sequences for the 23 Creole cattle from Guadeloupe samples are available from the European Nucleotide Archive (ENA) with the Bioproject Accession Number PRJEB58555.
