# Supplementary material for: Whole genome sequencing reveals signals of adaptive admixture in Creole cattle
Source: Sci Rep. 2023 Jul 27;13:12155. doi: 10.1038/s41598-023-38774-7 (PMC10374910; doi:10.1038/s41598-023-38774-7)
Supplement: Supplementary file 1 — Supplementary Information. [file 41598_2023_38774_MOESM1_ESM.pdf]

Whole genome sequencing reveals signals of adaptive admixture in Creole cattle

Supplementary material

Slim Ben-Jemaa[1,2*], Gabriele Adam[1], Mekki Boussaha[3], Philippe Bardou[4,5], Christophe Klopp[6], Nathalie Mandonnet[1] and Michel Naves[1*]

[1] INRAE, ASSET, 97170, Petit-Bourg, France.

[2] Laboratoire des Productions Animales et Fourragères, Institut National de la Recherche Agronomique de Tunisie, Université de Carthage, 2049 Ariana, Tunisia.

[3] Université Paris-Saclay, INRAE, AgroParisTech, GABI, 78350, Jouy-en-Josas, France.

[4] GenPhySE, Université de Toulouse, INRA, Ecole Nationale Vétérinaire de Toulouse (ENVT), 24 Chemin de Borde Rouge, 31320 Castanet-Tolosan,France

[5] Sigenae, INRAE, 24 Chemin de Borde Rouge, 31320 Castanet-Tolosan,France.

[6] Sigenae, Genotoul Bioinfo, BioInfoMics, MIAT UR875, INRAE, Castanet Tolosan, France

[*] Correspondence : Slim Ben Jemaa; Email : slim.ben-jemaa@inrae.fr/benjemaaslim@gmail.com

Michel Naves; Email : michel.naves@inrae.fr

(a) **(b)**

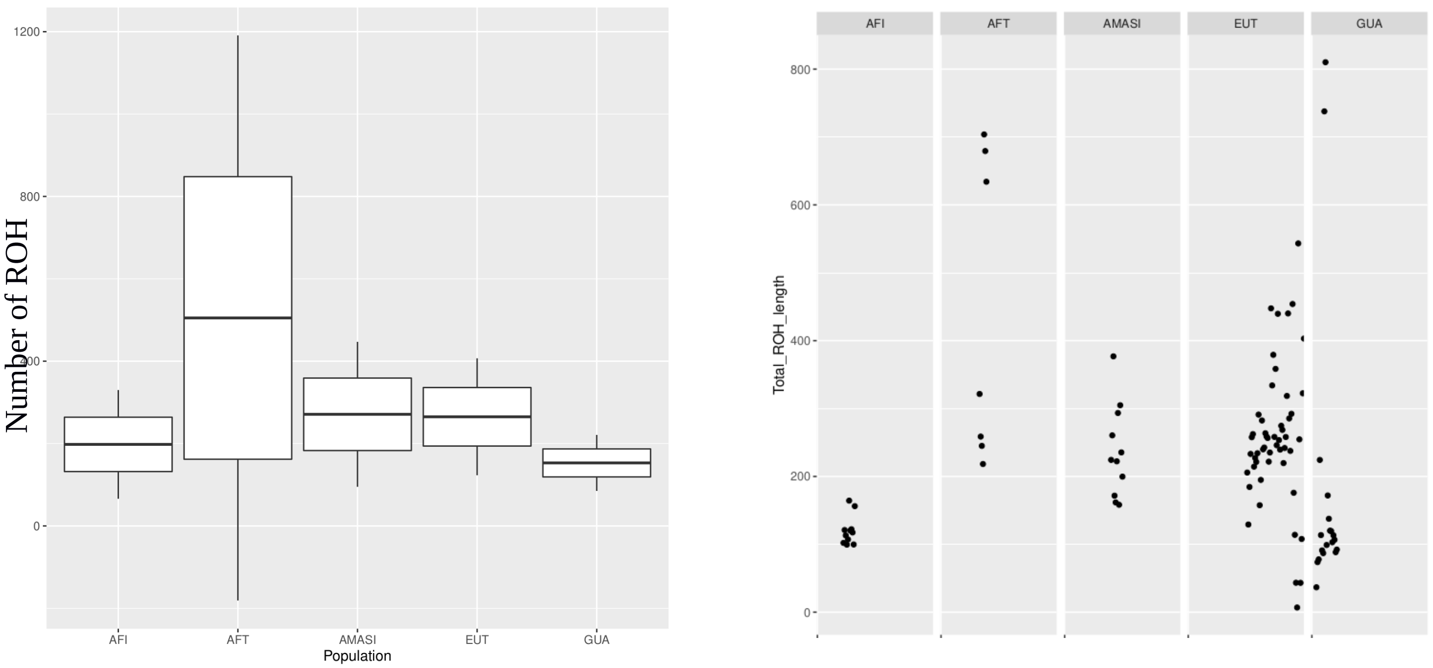

Supplementary Figure S1. ROH characterization. **(a)** Box plots of ROH number per group. **(b)** Cumulative ROH length (in Megabses) per individual. AFI : African indicine, AFT : African taurines , AMASI : American-Australian and Asian indicine, EUT : European taurines ; GUA : Creole cattle from Guandeloupe.

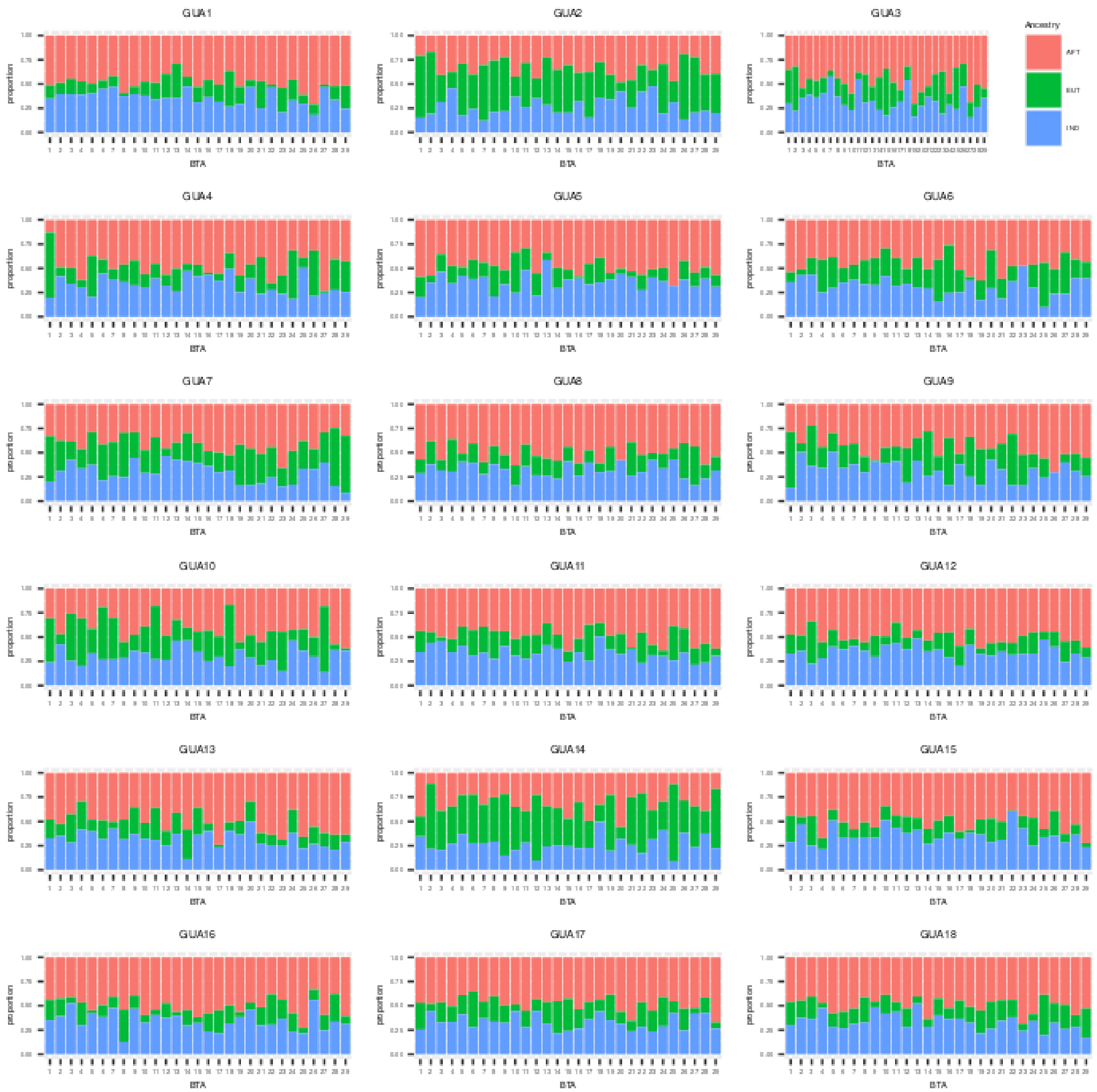

Supplementary Figure S2. Estimated local ancestries per GUA sample, across chromosomes, inferred from the ELAI software (red color for AFT, green color for EUT, blue color for IND).

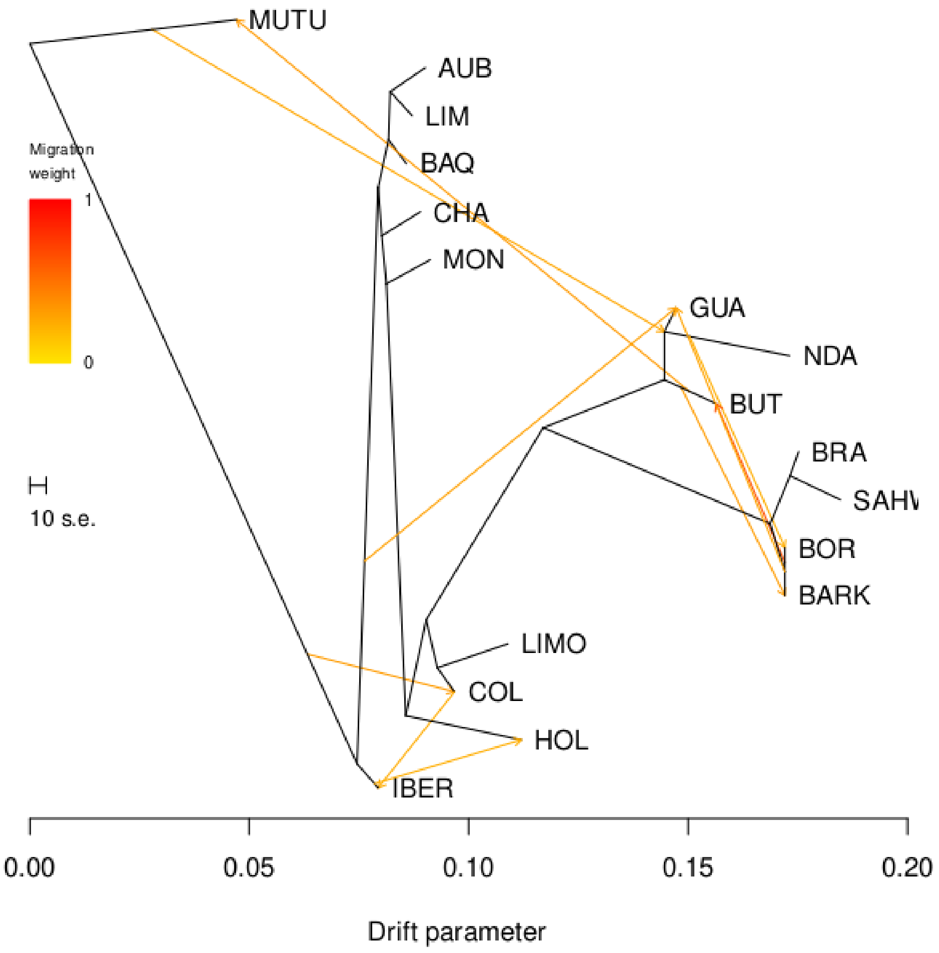

Supplementary Figure S3. Maximum likelihood tree constructed with TreeMix when 10 migration events (modeled as arrows) were allowed. Migration arrows are colored according to their weight.

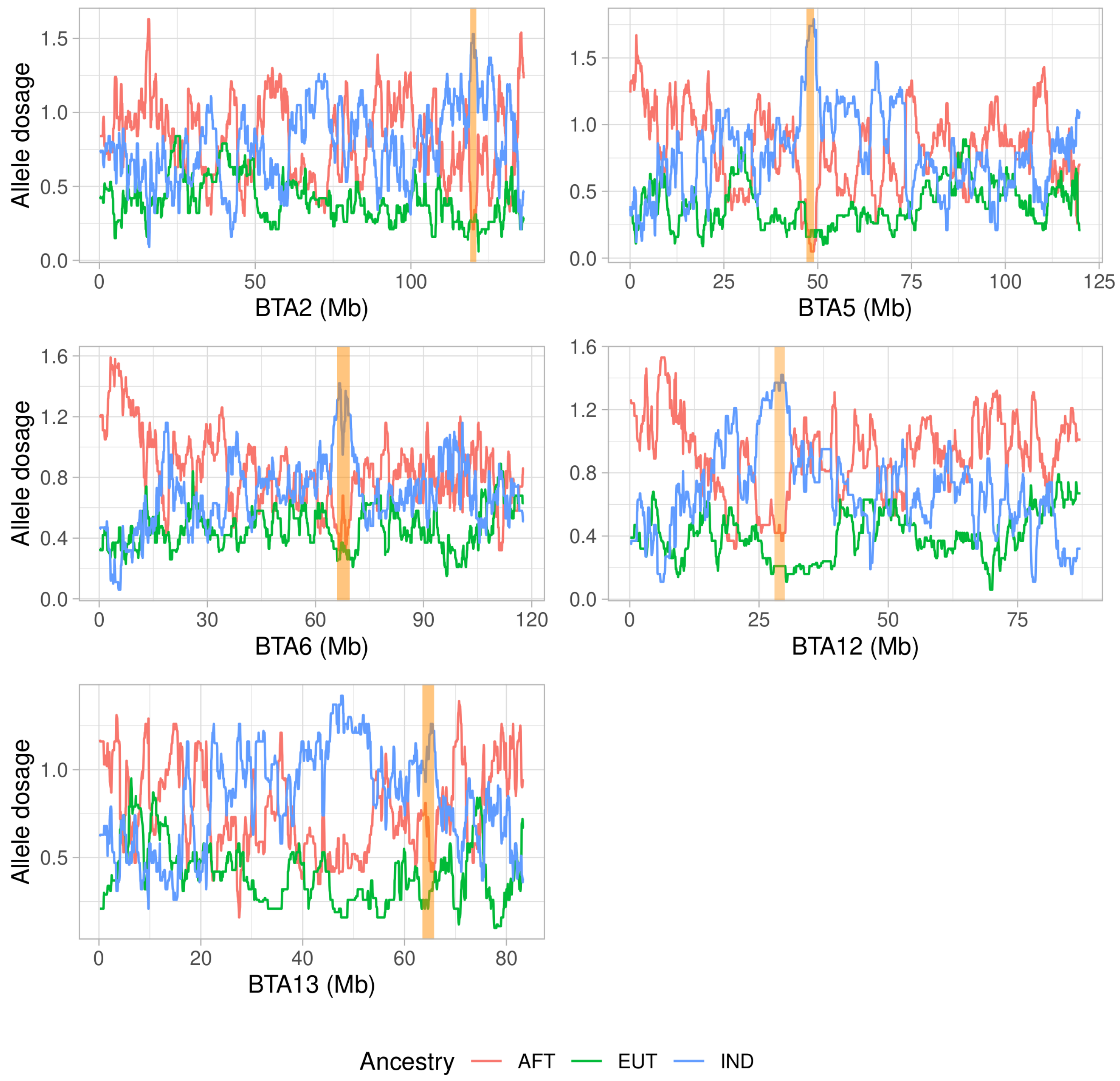

Supplementary Figure S4. Proportion of European taurines (EUT), African taurines (AFT) and zebu (IND) ancestries across the five candidate regions identified in the EUT Vs GUA comparison (*Rsb* and *XP-EHH* tests).

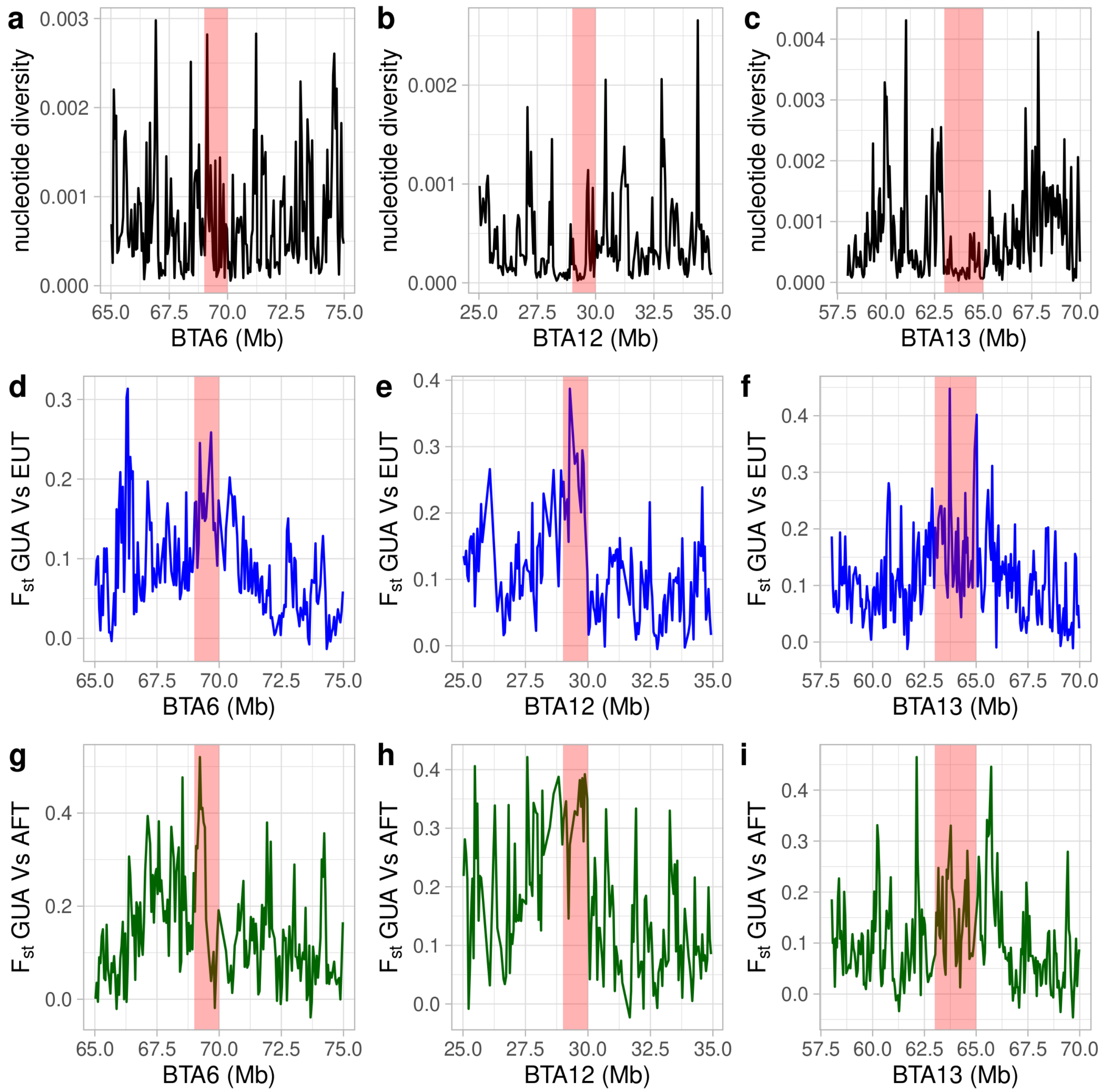

Supplementary Figure S5. Nucleotide diversity and Pairwise Fst values (EUT Vs GUA (blue) and AFT Vs GUA (green)) calculated for each 50-kb window around the candidate regions on chromosomes 6, 12 and 13. **(a)** nucleotide diversity on chromosome 6. **(b)** nucleotide diversity on chromosome 12. **(c)** nucleotide diversity on chromosome 13. **(d)** Pairwise Fst values between GUA and EUT on chromosome 6. **(e)** Pairwise Fst values between GUA and EUT on chromosome 12. **(f)** Pairwise Fst values between GUA and EUT on chromosome 13. **(g)** Pairwise Fst values between GUA and AFT on chromosome 6. **(h)** Pairwise Fst values between GUA and AFT on chromosome 12. **(i)** Pairwise Fst values between GUA and AFT on chromosome 13.

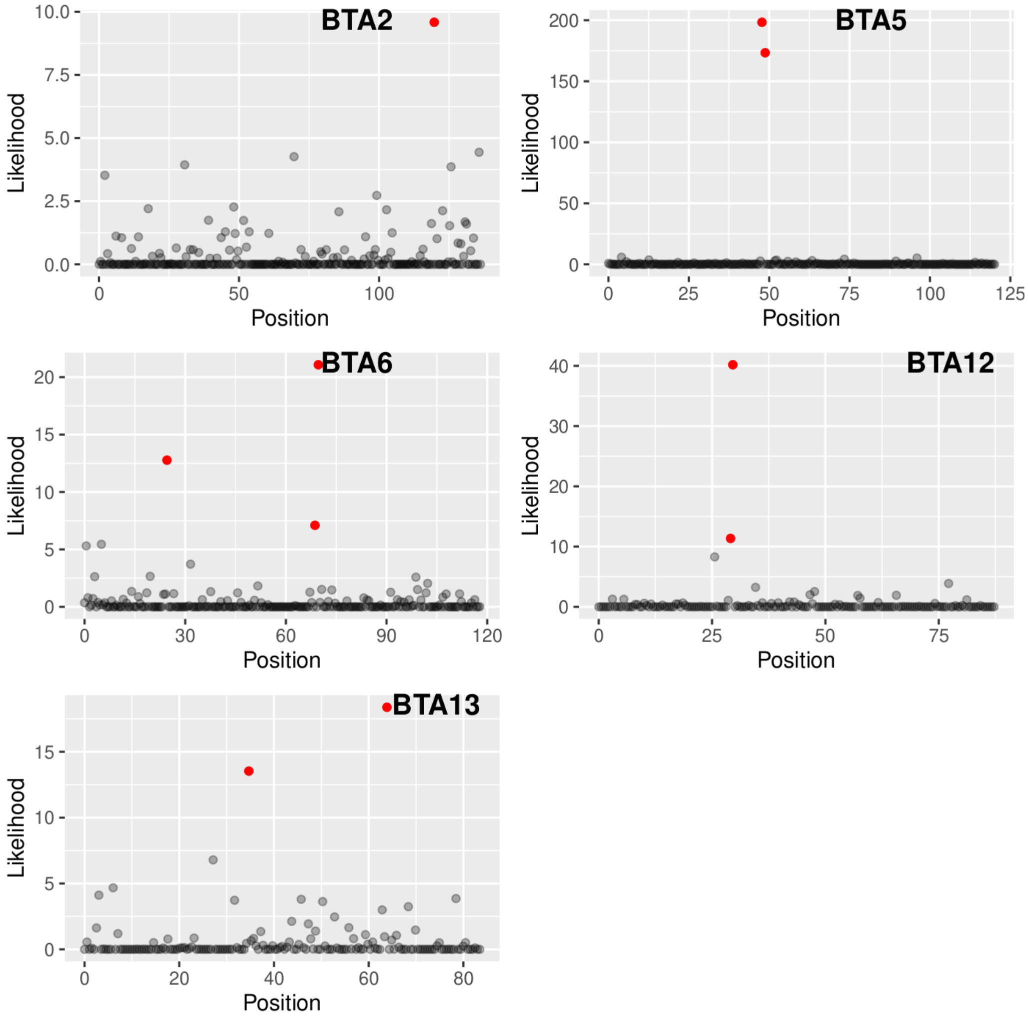

Supplementary Figure S6. Scan of the five genomic regions showing a local excess of indicine ancestry using SweeD. y axis shows the Composite Likelihood Ratio (CLR) in nonoverlapping 500-Kb windows along the chromosome. Red points correspond to the top outlier windows.

Supplementary Table S1. Summary of GUA sequencing data.

Sample_ID	Reads	Mapped reads	Mapping rate	Mapping depth	Sequencer
GUA20	321671142	308319167	95.85	16.08	HiSeq3000
GUA21	185963860	178672611	96.08	9.3	HiSeq3000
GUA22	190211772	181782828	95.57	10.34	HiSeq3000
GUA2	272926004	262009771	96.00	13.65	HiSeq3000
GUA1	419131126	401127757	95.70	20.96	HiSeq3000
GUA4	365286556	348179349	95.32	18.26	HiSeq3000
GUA3	213783080	204942141	95.86	10.69	HiSeq3000
GUA16	294066572	280454863	95.37	14.7	HiSeq3000
GUA6	403254024	385860397	95.69	20.16	HiSeq3000
GUA7	407586686	391938496	96.16	20.38	HiSeq3000
GUA8	268908996	257354278	95.7	13.45	HiSeq3000
GUA10	343876754	328435914	95.51	17.19	HiSeq3000
GUA13	355475416	339372280	95.47	17.77	HiSeq3000
GUA19	334272410	319796461	95.67	16.71	HiSeq3000
GUA5	368905906	353329064	95.78	18.45	HiSeq3000
GUA15	227384038	219455771	96.51	11.37	HiSeq3000
GUA9	475309462	451229948	94.93	23.77	HiSeq3000
GUA14	320407738	306296236	95.60	16.02	HiSeq3000
GUA11	366763352	349201156	95.21	18.34	HiSeq3000
GUA23	317963678	303253799	95.37	15.9	HiSeq3000
GUA17	372901582	358180879	96.05	18.65	HiSeq3000
GUA18	276537630	265481631	96.00	13.83	HiSeq3000
GUA12	413867220	396133238	95.72	20.69	HiSeq3000

Supplementary Table S2. Summary of SNPs discovered in the 23 Creole cattle from Guadeloupe individuals.

BTA	Chromsome length	SNPs	intermarker distance (bp)
1	158,534,110	1,053,161	150
2	136,231,102	944,752	144
3	121,005,158	802,650	150
4	120,000,601	861,206	139
5	120,089,316	829,619	144
6	117,806,340	828,782	142
7	110,682,743	762,351	145
8	113,319,770	715,924	158
9	105,454,467	720,569	146
10	103,308,737	717,829	143
11	106,982,474	718,007	148
12	87,216,183	653,449	133
13	83,472,345	577,043	144
14	82,403,003	533,810	154
15	85,007,780	653,112	130
16	81,013,979	535,056	151
17	73,167,244	494,940	147
18	65,820,629	457,991	143
19	63,449,741	398,821	159
20	71,974,595	528,250	136
21	69,862,954	460,875	151
22	60,773,035	393,619	154
23	52,498,615	430,881	121
24	62,317,253	435,114	143
25	42,350,435	283,567	149
26	51,992,305	360,180	144
27	45,612,108	344,314	132
28	45,940,150	338,621	135
29	51,098,607	394,490	129
Total/average	2,489,385,779	17,228,983	144

Supplementary Table S3. Distribution of SNPs identified in the 23 creole cattle from Guadeloupe individuals within the genomic regions annotated by snpEff.

Region/effect	Count	Percent
DOWNSTREAM	1,273,506	4.661%
EXON	242,844	0.889%
INTERGENIC	11,314,855	41.414%
INTRON	13,096,376	47.935%
SPLICE_SITE_ACCEPTOR	262	0.001%
SPLICE_SITE_DONOR	448	0.002%
SPLICE_SITE_REGION	22,934	0.084%
UPSTREAM	1,253,105	4.587%
UTR_3_PRIME	82,590	0.302%
UTR_5_PRIME	34,151	0.125%

Supplementary Table S4. Runs of homozygosity (ROH) islands in GUA. The Start and End columns indicate the coordinates of the consensus regions on the Bos taurus genome assembly ARS-UCD1.2. ROH islands in bold are overlapping or near the candidate regions detected in the selection signature analyses.

BTA	Start (bp)	END (bp)	Length (Kb)	% of animals
1	138,135,066	138169831	34.77	35
**5**	**47061785**	**47886925**	**825.14**	**35**
**5**	**48438356**	**49020572**	**582.22**	**41**
6	49337362	49345991	8.63	35
6	49414271	49437039	22.77	35
7	6252126	6549802	297.68	35
10	8763819	8879153	115.34	35
11	34764780	34919599	154.82	47
**12**	**28635496**	**28916516**	**281.02**	**41**
**12**	**29103153**	**29603209**	**500.06**	**35**
12	34316859	34521088	204.23	35
19	3151172	3346571	195.4	35

Supplementary Table S5. Fst values for each pair of populations represented by more than one individual, estimated from 1,064,342 autosomal SNPs using Genepop software. IBER population represents the four Spanish individuals. COL population represents the four Colombian individuals.

	GUA	LIMO	COL	BRA	SAHW	BUT	BARK	BOR	NDA	MUTU	IBER	AUB	CHA	BAQ	LIM	MON	HOL
GUA																	
LIMO	0.091																
COL	0.059	0.046															
BRA	0.105	0.238	0.220														
SAHW	0.106	0.254	0.227	0.052													
BUT	0.062	0.182	0.171	0.099	0.118												
BARK	0.050	0.167	0.156	0.066	0.080	0.033											
BOR	0.057	0.174	0.162	0.070	0.084	0.045	0.016										
NDA	0.100	0.202	0.175	0.283	0.308	0.217	0.192	0.202									
MUTU	0.148	0.253	0.212	0.378	0.408	0.312	0.282	0.292	0.227								
IBER	0.069	0.047	0.017	0.236	0.245	0.176	0.163	0.170	0.176	0.211							
AUB	0.086	0.090	0.058	0.240	0.247	0.197	0.183	0.189	0.195	0.227	0.051						
CHA	0.080	0.089	0.054	0.237	0.247	0.196	0.180	0.187	0.194	0.230	0.050	0.052					
BAQ	0.074	0.084	0.050	0.238	0.248	0.194	0.178	0.184	0.192	0.230	0.042	0.033	0.048				
LIM	0.078	0.087	0.053	0.234	0.242	0.191	0.177	0.183	0.189	0.224	0.044	0.035	0.049	0.029			
MON	0.081	0.090	0.056	0.238	0.246	0.193	0.179	0.185	0.196	0.231	0.050	0.055	0.055	0.052	0.052		
HOL	0.111	0.118	0.091	0.272	0.285	0.233	0.216	0.222	0.235	0.273	0.085	0.108	0.096	0.103	0.101	0.103	

Supplementary Table S6 Genomic regions putatively under selection identified using *iHS*, *Rsb* and *XP-EHH* statistics. Regions jointly identified by at least two methods are in bold.

Test	BTA	Start (Mb)	End (Mb)	Population under selection	Genes
iHS	**4**	**113**	**113.5**		**ENSBTAG00000053452, GIMAP4, GIMAP7, ENSBTAG00000040331, ENSBTAG00000011240, ENSBTAG00000054649, ENSBTAG00000050052, GIMAP7, , GIMAP5, ENSBTAG00000000715, TMEM176B, 5S_rRNA, TMEM176A, AOC1**
	**5**	**99**	**99.5**		**ENSBTAG00000052617, ENSBTAG00000052865, ENSBTAG00000053262, ENSBTAG00000046268, 7SK, ENSBTAG00000049367, ENSBTAG00000054018, KLRC1, ENSBTAG00000052486, ENSBTAG00000052514, ENSBTAG00000050324, KLRJ1, ENSBTAG00000051183, ENSBTAG00000049823**
RsbAFT Vs GUA	3	114.5	115	AFT	SH3BP4
	**4**	**112.5**	**113.5**	**AFT**	**ZNF746, ENSBTAG00000049343, KRBA1, ZNF467, SSPO, ZNF862, ATP6V0E2, LRRC61, RARRES2, REPIN1, ZNF775, ENSBTAG00000053931, ENSBTAG00000049318, GIMAP8, GIMAP7, ENSBTAG00000039588, GIMAP7, ENSBTAG00000053452, GIMAP4, GIMAP7, ENSBTAG00000040331, ENSBTAG00000011240, ENSBTAG00000054649, ENSBTAG00000050052, GIMAP7, ENSBTAG00000037510, GIMAP5, ENSBTAG00000000715, TMEM176B, 5S_rRNA, TMEM176A, AOC1**
	**5**	**99**	**99.5**	**GUA**	**ENSBTAG00000052617, ENSBTAG00000052865, ENSBTAG00000053262, ENSBTAG00000046268, 7SK, ENSBTAG00000049367, ENSBTAG00000054018, KLRC1, ENSBTAG00000052486, ENSBTAG00000052514, ENSBTAG00000050324, KLRJ1, ENSBTAG00000051183, ENSBTAG00000049823**
	11	100.5	101	AFT	NCS1, HMCN2, ASS1, FUBP3, U6, PRDM12, EXOSC2, ABL1
	**20**	**71.5**	**72**	**AFT**	**CEP72, SLC9A3, EXOC3, ENSBTAG00000026527, AHRR, 5S_rRNA, PDCD6, SDHA, LRRC14B, CCDC127, U6, ENSBTAG00000055240, ENSBTAG00000048135, ENSBTAG00000047700, ENSBTAG00000047632**
	28	38	38.5	AFT	NRG3
Rsb EUT Vs GUA	**1**	**76.5**	**77**	**EUT**	**IL1RAP, TMEM207, CLDN16, CLDN1**
	2	5.5	6	EUT	ENSBTAG00000017214, NAB1, NEMP2, MFSD6, INPP1, HIBCH
	**2**	**120**	**120.5**	**GUA**	**ENSBTAG00000053448, ENSBTAG00000016748, ENSBTAG00000051665, ENSBTAG00000039346, ALPI, ENSBTAG00000012363, ECEL1, ENSBTAG00000050396, PRSS56, CHRND, CHRNG, EIF4E2,**

					**PHC2, ENSBTAG00000054666, ZNF362**
	**5**	**47**	**47.5**	**GUA**	**GRIP1, U1, HELB**
	**5**	**74**	**74.5**	**EUT**	**RBFOX2, ENSBTAG00000046392, ENSBTAG00000049297, ENSBTAG00000037799, ENSBTAG00000052884, ENSBTAG00000055135, ENSBTAG00000053500, ENSBTAG00000048740, ENSBTAG00000053144, ENSBTAG00000050417**
	**6**	**69**	**69.5**	**GUA**	**FIP1L1,LNX1, ENSBTAG00000004082**
	**12**	**29**	**30**	**GUA**	**RXFP2, ENSBTAG00000053332, bta-mir-2299, B3GLCT, HSPH1, ENSBTAG00000016052, ENSBTAG00000053517, TEX26, MEDAG**
	**13**	**63.5**	**64**	**GUA**	**ASIP, AHCY, ENSBTAG00000050108, ENSBTAG00000046623, ITCH, DYNLRB1, MAP1LC3A, PIGU, ASIP**
	**15**	**50**	**50.5**	**EUT**	**ENSBTAG00000051323, ENSBTAG00000049986, OR51G2, U6, ENSBTAG00000050365, OR51H9B, OR51H5, OR51S1B, OR51A8, ENSBTAG00000038578, OR52R1E, OR51F1B, OR51F5C, OR51F5B, OR51F23D, OR51E2, OR51A25, OR51A57, OR51A49, OR51E1, OR51D1, TRIM68, OR52I1B, OR52I1, OR52I11**
	18	58	58.5	EUT	ENSBTAG00000052289, PPP2R1A, 5S_rRNA, ENSBTAG00000046864, ENSBTAG00000018162, ENSBTAG00000050064, ENSBTAG00000049460, ENSBTAG00000054547, ENSBTAG00000050488, ENSBTAG00000054038, ENSBTAG00000047761, ENSBTAG00000053131, ENSBTAG00000038903, ENSBTAG00000051725, ENSBTAG00000033523, ENSBTAG00000049736, ENSBTAG00000055293, ENSBTAG00000017651, ENSBTAG00000011052
	**18**	**61**	**61.5**	**EUT**	**MGC157082, ENSBTAG00000014953, ENSBTAG00000000336, ENSBTAG00000009171, ENSBTAG00000015061, MGC138914, ENSBTAG00000054918, ENSBTAG00000013345, ENSBTAG00000009364, ENSBTAG00000015987, bta-mir-11977, ENSBTAG00000051856, ENSBTAG00000046961, ENSBTAG00000051149, ENSBTAG00000030416, MGC157082, ENSBTAG00000014953, ENSBTAG00000000336, ENSBTAG00000009171, ENSBTAG00000015061, MGC138914, ENSBTAG00000054918, ENSBTAG00000013345, ENSBTAG00000009364, ENSBTAG00000015987, bta-mir-11977**
Rsb IND Vs GUA	**1**	**26**	**26.5**	**IND**	**ROBO1**
	2	12.5	13	IND	ENSBTAG00000042196, ENSBTAG00000054621, ENSBTAG00000052131
	3	40	40.5	IND	COL11A1
	**5**	**99**	**99.5**	**GUA**	**ENSBTAG00000052617, ENSBTAG00000052865, ENSBTAG00000053262, ENSBTAG00000046268, 7SK, ENSBTAG00000049367, ENSBTAG00000054018, KLRC1, ENSBTAG00000052486, ENSBTAG00000052514, ENSBTAG00000050324, KLRJ1, ENSBTAG00000051183,**

					**ENSBTAG00000049823**
	**7**	**105**	**105.5**	**IND**	**No genes**
	**13**	**83**	**83.5**	**IND**	**CBLN4**
	**21**	**13**	**13.5**	**IND**	**ENSBTAG00000033335**
	**27**	**30.5**	**31**	**IND**	**ENSBTAG00000042113, UNC5D, ENSBTAG00000005639**
XP-EHH AFT Vs GUA	**4**	**112.5**	**114**	**AFT**	**ZNF746, ENSBTAG00000049343, KRBA1, ZNF467, SSPO, ZNF862, ATP6V0E2, LRRC61, RARRES2, REPIN1, ZNF775, ENSBTAG00000053931, ENSBTAG00000049318, GIMAP8, GIMAP7, ENSBTAG00000039588, GIMAP7, ENSBTAG00000053452, GIMAP4, GIMAP7, ENSBTAG00000040331, ENSBTAG00000011240, ENSBTAG00000054649, ENSBTAG00000050052, GIMAP7, ENSBTAG00000037510, GIMAP5, ENSBTAG00000000715, TMEM176B, 5S_rRNA, TMEM176A, AOC1, KCNH2, NOS3, ATG9B, ABCB8, ASIC3, CDK5, SLC4A2, FASTK, bta-mir-6525, TMUB1, ENSBTAG00000048379, AGAP3, ASB10, GBX1, IQCA1L, H2BK1, ABCF2, CHPF2, bta-mir-671, SMARCD3, ENSBTAG00000048514, NUB1, WDR86, CRYGN**
	**5**	**99**	**99.5**	**GUA**	**ENSBTAG00000052617, ENSBTAG00000052865, ENSBTAG00000053262, ENSBTAG00000046268, 7SK, ENSBTAG00000049367, ENSBTAG00000054018, KLRC1, ENSBTAG00000052486, ENSBTAG00000052514, ENSBTAG00000050324, KLRJ1, ENSBTAG00000051183, ENSBTAG00000049823**
	**20**	**71.5**	**72**	**AFT**	**CEP72, SLC9A3, EXOC3, ENSBTAG00000026527, AHRR, 5S_rRNA, PDCD6, SDHA, LRRC14B, CCDC127, U6, ENSBTAG00000055240, ENSBTAG00000048135, ENSBTAG00000047700, ENSBTAG00000047632**
XP-EHH EUT Vs GUA	**1**	**76.5**	**77**	**EUT**	**IL1RAP, TMEM207, CLDN16, CLDN1**
	**2**	**120**	**120.5**	**GUA**	**ENSBTAG00000053448, ENSBTAG00000016748, ENSBTAG00000051665, ENSBTAG00000039346, ALPI, ENSBTAG00000012363, ECEL1, ENSBTAG00000050396, PRSS56, CHRND, CHRNG, EIF4E2, PHC2, ENSBTAG00000054666, ZNF362**
	**5**	**47**	**47.5**	**GUA**	**GRIP1, U1, HELB**
	5	48.5	49	GUA	MSRB3, LEMD3, WIF1, U6, TBC1D30
	**5**	**74**	**74.5**	**EUT**	**RBFOX2, ENSBTAG00000046392, ENSBTAG00000049297, ENSBTAG00000037799, ENSBTAG00000052884, ENSBTAG00000055135, ENSBTAG00000053500, ENSBTAG00000048740, ENSBTAG00000053144, ENSBTAG00000050417**
	**6**	**69**	**69.5**	**GUA**	**FIP1L1,LNX1, ENSBTAG00000004082**
	9	8	8.5		ADGRB3, ENSBTAG00000054672

	10	99	99.5		No genes
	**12**	**29**	**30**	**GUA**	**RXFP2, ENSBTAG00000053332, bta-mir-2299, B3GLCT, HSPH1, ENSBTAG00000016052, ENSBTAG00000053517, TEX26, MEDAG**
	12	61.5	62		No genes
	**13**	**63.5**	**64**	**GUA**	**ASIP, AHCY, ENSBTAG00000050108, ENSBTAG00000046623, ITCH, DYNLRB1, MAP1LC3A, PIGU, ASIP**
	**15**	**50**	**50.5**	**EUT**	**ENSBTAG00000051323, ENSBTAG00000049986, OR51G2, U6, ENSBTAG00000050365, OR51H9B, OR51H5, OR51S1B, OR51A8, ENSBTAG00000038578, OR52R1E, OR51F1B, OR51F5C, OR51F5B, OR51F23D, OR51E2, OR51A25, OR51A57, OR51A49, OR51E1, OR51D1, TRIM68, OR52I1B, OR52I1, OR52I11**
	**18**	**61**	**61.5**	**EUT**	**MGC157082, ENSBTAG00000014953, ENSBTAG00000000336, ENSBTAG00000009171, ENSBTAG00000015061, MGC138914, ENSBTAG00000054918, ENSBTAG00000013345, ENSBTAG00000009364, ENSBTAG00000015987, bta-mir-11977, ENSBTAG00000051856, ENSBTAG00000046961, ENSBTAG00000051149, ENSBTAG00000030416, MGC157082, ENSBTAG00000014953, ENSBTAG00000000336, ENSBTAG00000009171, ENSBTAG00000015061, MGC138914, ENSBTAG00000054918, ENSBTAG00000013345, ENSBTAG00000009364, ENSBTAG00000015987, bta-mir-11977**
XP-EHH IND Vs GUA	**1**	**26**	**26.5**	**IND**	**ROBO1**
	**5**	**99**	**99.5**	**GUA**	**ENSBTAG00000052617, ENSBTAG00000052865, ENSBTAG00000053262, ENSBTAG00000046268, 7SK, ENSBTAG00000049367, ENSBTAG00000054018, KLRC1, ENSBTAG00000052486, ENSBTAG00000052514, ENSBTAG00000050324, KLRJ1, ENSBTAG00000051183, ENSBTAG00000049823**
	**7**	**105**	**105.5**	**IND**	**No genes**
	**13**	**83**	**83.5**	**IND**	**CBLN4**
	14	59,5	60	IND	ENSBTAG00000042347
	15	3,5	4	IND	ENSBTAG00000051613, ENSBTAG00000052675
	**21**	**13**	**13.5**	**IND**	**ENSBTAG00000033335**
	**27**	**30.5**	**31**	**IND**	**ENSBTAG00000042113, UNC5D, ENSBTAG00000005639**

Supplementary Table S7. Top 1% regions with the highest indicine ancestry across the GUA genome. Regions overlapping with the four candidate windows on chromosomes 2, 5, 6, 12 and the region located nearby the candidate window on chromosome 13, detected in the GUA Vs EUT comparison, are in bold.

Chromsome	Region (Mb)	Average allele dosage of indicine ancestry
**2**	**119.08 - 121.125**	**1.44**
2	124.68 – 126.075	1.33
4	62.075 - 63.475	1.34
**5**	**46.93 – 49.725**	**1.6**
5	64.83 - 66.725	1.38
**6**	**65.975 - 69.775**	**1.32**
7	21.03 - 25.825	1.28
9	70.075 - 71.225	1.32
9	74.23 - 75.775	1.34
10	15.63 - 17.325	1.36
10	30.73 - 31.175	1.29
11	35.825 - 36.175	1.29
**12**	**27.575 – 30.125**	**1.36**
13	45.575 - 48.125	1.37
**13**	**65.175 - 65.775**	**1.27**
18	57.225 - 58.775	1.33

Supplementary Table S8. Top 1% regions with the highest Fst between GUA and EUT. Regions overlapping with the five candidate windows on chromosomes 2, 5, 6, 12 and 13 detected in the GUA Vs EUT comparison are in bold.

Chromosome	Region (Mb)	N SNPs	Average Fst GUA/EUT
2	4.925 - 6.325	67	0.26
2	72.725 - 76.52	60	0.3
**2**	**119.125 - 120.475**	**49**	**0.31**
2	124.375 - 125.675	39	0.28
3	73.475 - 79.175	89	0.3
4	63.225 - 67.075	68	0.28
5	41.775 - 42.275	64	0.27
**5**	**46.225 - 49.225**	**219**	**0.34**
5	51.475 - 54.375	60	0.28
5	59.825 - 61.225	52	0.27
5	65.675 - 68.925	55	0.27
5	103.32 - 106.975	77	0.28
**6**	**66.275 - 69.675**	**30**	**0.29**
8	104.875 - 106.475	52	0.3
9	63.225 - 66.875	38	0.3
10	17.125 - 21.875	115	0.29
10	29.975 - 32.175	40	0.28
10	71.075 - 74.325	70	0.29
11	38.725 - 40.575	53	0.31
**12**	**29.275 - 29.825**	**37**	**0.3**
13	47.225 - 50.225	80	0.3
**13**	**62.875 - 65.775**	**77**	**0.27**
16	41.225 - 45.875	30	0.28
21	30.425 - 33.075	83	0.32
23	0.175 - 1.025	70	0.28
23	11.625 - 15.575	59	0.28

Supplementary Table S9. Top 1% regions with the highest Fst between GUA and AFT. Regions overlapping with the five candidate windows on chromosomes 2, 5, 6, 12 and 13 detected in the GUA Vs EUT comparison are in bold.

Chromosome	Region (Mb)	N SNPs	Average Fst GUA/EUT
2	43.68 - 47.675	53	0.34
2	61.93 - 63.475	37	0.36
2	72.33 - 77.375	164	0.37
**2**	**119.98 - 122.825**	**56**	**0.42**
2	125.53 - 126.22	88	0.4
3	11.78 - 16.525	51	0.4
3	18.93 - 19.175	46	0.37
3	37.03 - 38.575	98	0.37
3	58.28 - 62.225	94	0.35
**5**	**45.68 - 50.325**	**292**	**0.43**
5	52.28 - 55.625	126	0.39
5	56.63 - 59.475	38	0.43
6	61.13 - 62.425	68	0.38
**6**	**67.13 - 71.925**	**183**	**0.4**
9	0.78 - 3.475	82	0.41
10	30.93 - 31.275	52	0.41
10	47.98 - 49.875	100	0.37
11	26.38 - 28.625	49	0.39
11	36.03 - 39.575	111	0.36
12	19.63 - 20.525	54	0.38
12	24.03 - 26.825	52	0.36
**12**	**27.575 - 31.925**	**203**	**0.36**
12	36.725 - 38.625	145	0.4
**13**	**62.125 - 65.725**	**32**	**0.42**
15	77.325 - 79.925	211	0.39
17	51.125 - 51.325	45	0.4
18	58.275 - 59.225	71	0.36
20	18.575 - 19.275	65	0.35
20	23.875 - 25.875	48	0.37
21	54.375 - 55.475	162	0.38
21	60.175 - 62.975	115	0.36

27	45.325 - 45.625	148	0.45

Supplementary Table S10. Genomic regions under selection in AFT detected by *Rsb AFT Vs EUT*

Chromosome	Start(Mb)	End (Mb)	N SNPs	N extreme SNPs
4	112.5	113.5	1122	37
5	10	10.5	155	5
5	74	74.5	70	13
6	66.5	67	80	5
14	49	49.5	79	5
20	71.5	72	56	9

Supplementary Table S11. Genomic regions under selection in AFT detected by *Rsb AFT Vs IND*

Chromosome	Start(Mb)	End (Mb)	N SNPs	N extreme SNPs
4	112.5	113.5	1122	46
20	71.5	72	64	7

Supplementary Table S12. Candidate regions overlapping with those reported in Gautier & Naves, 2011 when we used the same criteria as these authors (278 regions are identified in total). Only *Rsb EUT Vs GUA* results are reported. Rows in bold indicate a position mismatch between the two studies likely owing to differences in genome assemblies used in the two studies.

Chromosome	START(Mb)	END(Mb)	N SNPs	N extreme SNPs	Region reported in Gautier & Naves
2	129.5	131	279	1	
3	107.5	110	327	8	
**5**	**22.5**	**24**	**237**	**1**	**27.8–28.8**
5	60.5	62	168	3	
**5**	**66**	**67.5**	**108**	**6**	**64.0–65.1**
7	29	30.5	298	2	
**7**	**48**	**49.5**	**182**	**1**	**52.1–53.1**
10	30.5	34.5	558	7	
**11**	**54.5**	**56**	**178**	**1**	**50.6–51.7**
12	28	31	293	64	
13	45.5	47	158	3	
13	58	59.5	223	3	
**18**	**14**	**17**	**346**	**14**	**12.3–13.3**

Supplementary Table S13. Sample description. The number in the bracket in the Number of individuals column corresponds to the number of individuals after relatedness filtering.

Population name	Abbreviation	Country	Number	Type†	Data origin
Guadeloupe cattle	GUA	France	23 (19)		This study
Barka	BARK	Ethiopia	4(4)	AFI	Kim et al., 2020
Boran	BOR	Ethiopia	4(4)	AFI	Kim et al., 2020
Butana	BUT	Sudan	4(3)	AFI	Kim et al., 2020
Muturu	MUTU	Nigeria	3(3)	AFT	Tijjani et al., 2019[1]
N'Dama	NDA	Gambia & Guinea	7(4)	AFT	Kim et al., 2020
Brahman	BRA	Australia	5(5)	AMI	Koufariotis et al., 2018[2]
Gir	GIR	Unites states	1(1)	AMI	PRJNA277147
Nelore	NEL	Unites states	1(1)	AMI	PRJNA277147
Hariana	HAR	India	1(1)	ASI	Chen et al., 2018
Sahiwal	SAHW	Pakistan	3(3)	ASI	Talenti et al., 2022[3]
Blanco Orejinegro	COL	Colombia	1(1)	CRIO	IMAGE project
Romosinuano	COL	Colombia	1(1)	CRIO	IMAGE project
Costeño con cuernos	COL	Colombia	1(1)	CRIO	IMAGE project
San Martinero	COL	Colombia	1(1)	CRIO	IMAGE project
Limonero	LIMO	Venezuela	4(4)	CRIO	Port-Neto et al., 2018[4]
Limia	IBER	Spain	1(1)	EUT	Upadhyay et al., 2019[5]
Pajuna	IBER	Spain	1(1)	EUT	Upadhyay et al., 2019[5]
Sayaguesa	IBER	Spain	1(1)	EUT	Upadhyay et al., 2019[5]
Maronesa	IBER	Spain	1(1)	EUT	Upadhyay et al., 2019[5]
Aubrac	AUB	France	8(8)	EUT	The 1000 Bull Genomes Project

blonde d'Aquitaine	BAQ	France	8(5)	EUT	The 1000 Bull Genomes Project
Charolais	CHA	France	7(7)	EUT	The 1000 Bull Genomes Project
Holstein	HOL	France	8(6)	EUT	The 1000 Bull Genomes Project
Limousin	LIM	France	9(7)	EUT	The 1000 Bull Genomes Project
Montbeliarde	MON	France	10(6)	EUT	The 1000 Bull Genomes Project
Total			118(99)		

† AFI : African indicine ; AFT : African taurine ; AMI : Australian-American indicine; ASI : Asian indicine ; CRIO : Criollo ; EUT : European taurine

Supplementary Table S14. Marker statistics by chromosome for the 1,064,342 SNPs retained after quality control filters.

BTA	SNPs	Mean intermarker distance (bp)	Median intermarker distance (bp)	Minimum intermarker distance (bp)	Maximum intermarker distance (bp)
1	62647	2530±4317	1138	1	141808
2	51055	2667±4904	1157	1	333617
3	46982	2576±4616	1101	1	165677
4	50985	2351±4296	976	1	127531
5	46465	2584±4780	1035	1	167448
6	48340	2437±4009	1143	1	93029
7	45035	2456±5140	1012	1	353252
8	41340	2739±4849	1205	1	154152
9	43222	2420±4102	1052	1	98939
10	44546	2318±6566	875	1	1015085
11	39483	2709±4600	1188	1	94933

12	41956	2078±4164	790	1	146566
13	33583	2485±4682	1023	1	182993
14	33674	2446±4493	1050	1	161265
15	40133	2117±4060	872	1	180689
16	34427	2351±4956	985	1	242602
17	33248	2199±3937	984	1	185377
18	31065	2119±4264	759	1	251881
19	27274	2325±4757	904	1	150887
20	31305	2299±4145	1029	1	123939
21	28447	2455±5006	953	1	243166
22	26185	2320±4101	988	1	109355
23	39208	1339±2868	423	1	106882
24	27547	2262±3862	994	1	110379
25	20556	2060±3504	890	1	74316
26	23614	2199±4266	962	1	231611
27	23700	1924±3205	850	1	78376
28	22163	2073±3589	905	1	92397
29	26157	1953±3498	831	1	78543
Total/mean	1064342	2303±4329			

Supplementary references

1. Tijjani, A., Utsunomiya, Y. T., Ezekwe, A. G., Nashiru, O. & Hanotte, O. Genome Sequence Analysis Reveals Selection Signatures in Endangered Trypanotolerant West African Muturu Cattle. *Front. Genet.* **10**, (2019).
2. Koufariotis, L. *et al.* Sequencing the mosaic genome of Brahman cattle identifies historic and recent introgression including polled. *Sci Rep* **8**, 17761 (2018).
3. Talenti, A. *et al.* A cattle graph genome incorporating global breed diversity. *Nat Commun* **13**, 910 (2022).
4. Porto-Neto, L. R. *et al.* Convergent Evolution of Slick Coat in Cattle through Truncation Mutations in the Prolactin Receptor. *Frontiers in Genetics* **9**, (2018).
5. Upadhyay, M. *et al.* Deciphering the patterns of genetic admixture and diversity in southern European cattle using genome-wide SNPs. *Evolutionary Applications* **12**, 951–963 (2019).
